# FAPα^+^ Macrophages Orchestrate Immune Evasion in Multiple Myeloma by Dual Regulation of PD‐L1 and T Cell Senescence

**DOI:** 10.1002/advs.202506239

**Published:** 2026-01-30

**Authors:** Huiyao Gu, Zhenfeng Dai, Xi Huang, Enfan Zhang, Xinyuan Dai, Haoguang Chen, Wen Cao, Jinna Zhang, Yifan Hou, Haimeng Yan, Yang Yang, Li Yang, Yi Li, Wenlong Lin, Zhen Cai, Jingsong He

**Affiliations:** ^1^ Bone Marrow Transplantation Center the First Affiliated Hospital Zhejiang University School of Medicine Hangzhou Zhejiang China; ^2^ Institute of Immunology and the Second Affiliated Hospital Zhejiang University Hangzhou China; ^3^ Institute of Hematology Zhejiang University Hangzhou Zhejiang China

**Keywords:** FAPα, immune escape, immunotherapy, macrophage, multiple myeloma, PD‐L1, vimentin

## Abstract

Multiple myeloma (MM) is a hematologic malignancy driven by clonal expansion of malignant plasma cells. Despite the long‐term disease control achieved with immunotherapies in some patients, treatment resistance remains a major cause of disease relapse. Accumulating evidence highlights the tumor immune microenvironment, especially macrophages, as a key contributor to immunotherapy failure in MM. Herein, we identified a subset of MM‐associated macrophages with high expression of fibroblast activation protein alpha (FAPα), defined as FAPα^+^ macrophages. Clinical data showed that FAPα^+^ macrophages were enriched in the bone marrow versus peripheral blood of MM patients, and their abundance positively correlated with tumor burden. In MM mouse models, depletion of FAPα^+^ macrophages significantly boosted the efficacy of anti‐PD‐1/PD‐L1 antibody therapy but not anti‐CTLA‐4 therapy; this combinatorial strategy also exerted enhanced anti‐tumor effects in EL4 lymphoma and CT26 colorectal carcinoma models. Mechanistically, FAPα stabilized PD‐L1 expression by maintaining its N‐glycosylation and inhibiting proteasomal degradation, and induced PD‐L1 synthesis via promoting vimentin (VIM) phosphorylation at the S72 residue. Additionally, FAPα^+^ macrophages accelerated T cell senescence by secreting soluble FAPα. Collectively, our findings demonstrate that FAPα^+^ macrophages mediate MM immune evasion via dual mechanisms, positioning them as promising therapeutic targets to potentiate anti‐tumor immunotherapies.

## Graphical abstract

Fibroblast activation protein alpha‐positive (FAPα^+^) macrophages, a distinct subset of multiple myeloma (MM)‐associated macrophages, drive immune evasion in MM through multi‐faceted mechanisms. FAPα physically interacts with vimentin (VIM) and triggers its phosphorylation at the S72 residue, which in turn induces PD‐L1 transcription. Additionally, FAPα sustains PD‐L1 protein stability by preserving its N‐glycosylation and suppressing proteasomal degradation of PD‐L1. Moreover, FAPα^+^ macrophages secrete soluble FAPα to accelerate T cell senescence, further facilitating MM immune escape. Collectively, these findings identify FAPα^+^ macrophages as a key mediator of MM immune evasion and a promising therapeutic target for developing novel anti‐MM immunotherapies.

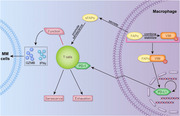

## 1 Introduction

Multiple myeloma (MM) is a hematologic malignancy with a steadily rising incidence worldwide. Historically, first‐line therapeutic strategies for MM have centered on proteasome inhibitors (PIs) and immunomodulatory drugs (IMiDs) [1]. The advent of immunotherapies, particularly chimeric antigen receptor T (CAR‐T) cell therapy, has revolutionized the treatment landscape of MM, ushering in a new era of clinical management [2]. Despite this breakthrough, a considerable proportion of MM patients eventually develop resistance to CAR‐T cell therapy, severely limiting the long‐term efficacy of this treatment. Immune checkpoint inhibitors (ICIs), including antibodies targeting programmed cell death protein 1 (PD‐1) and programmed death‐ligand 1 (PD‐L1), have demonstrated striking therapeutic benefits in subsets of solid tumors, yet their efficacy in hematologic malignancies such as MM has been notably disappointing [3, 4]. Notably, a recent study reported that CAR‐T cells engineered with PD‐1 downregulation exhibited enhanced anti‐tumor activity in MM [5]. These observations underscore the urgent need to further explore combination therapies incorporating anti‐PD‐1/PD‐L1 antibodies (αPD‐1/αPD‐L1) and to elucidate the underlying mechanisms governing their synergistic or antagonistic effects.

Dysregulated immune surveillance is a well‐recognized hallmark of tumorigenesis and progression. The tumor microenvironment (TME) is a complex ecosystem composed of a diverse array of immune cells, including tumor‐associated macrophages (TAMs) [6, 7], T cells [8], B cells [9], and granulocytes [10]. Among these cellular components, TAMs are distinguished by their extraordinary phenotypic and functional plasticity, emerging as key regulators of tumor biology and thus attracting growing research attention. Accumulating evidence suggests that PD‐L1 expressed on the surface of TAMs, rather than on tumor cells themselves, plays a dominant role in mediating tumor immune evasion [11], although the precise molecular mechanisms underlying this process remain largely elusive.

Cancer‐associated fibroblasts (CAFs) are known to promote tumor metastasis and chemoresistance [12, 13]; recent studies further implicate CAFs in the induction of tumor immune tolerance and even the attenuation of CAR‐T cell therapy efficacy [14, 15, 16]. However, the molecular pathways linking CAFs to immune suppression in the TME are poorly characterized. Fibroblast activation protein α (FAPα), a well‐established biomarker of activated fibroblasts, is widely used to monitor disease progression in solid tumors [14, 15, 16, 17, 18]. Nevertheless, the mechanisms through which FAPα contributes to tumorigenesis remain incompletely understood. Beyond its canonical expression on activated fibroblasts, FAPα has also been detected in other cell types, including endothelial cells, pericytes, and TAMs [19, 20, 21]. TAMs constitute a major cellular component of the TME in various malignancies, with a particularly prominent presence in MM [22, 23]. Despite extensive research on TAMs, macrophage heterogeneity extending beyond the classical M1/M2 polarization paradigm remains inadequately defined [24, 25]. Importantly, the existence and functional significance of FAPα^+^ TAMs in MM patients have not been previously investigated.

In this study, we identified a distinct subset of MM‐associated macrophages characterized by high FAPα expression, which we designated FAPα^+^ macrophages. Clinical analyses revealed that FAPα^+^ macrophages are markedly enriched in the bone marrow (BM) compared to the peripheral blood of MM patients, and they account for a larger proportion of cells than FAPα^+^ bone marrow stromal cells (BMSCs) within the BM niche. Moreover, the abundance of FAPα^+^ macrophages was positively correlated with MM tumor burden. In tumor‐bearing mouse models, combination therapy with αPD‐1/αPD‐L1 antibodies and FAPα^+^ macrophage blockade significantly improved treatment responses not only in MM but also in EL4 T‐lymphoma and CT26 colon carcinoma models. Mechanistically, FAPα interacts with vimentin (VIM) and promotes its phosphorylation at the S72 residue, thereby inducing PD‐L1 expression; concurrently, FAPα maintains PD‐L1 protein stability by preserving its N‐glycosylation modification and inhibiting proteasomal degradation. Furthermore, we demonstrated that FAPα^+^ macrophages accelerate T cell senescence via the secretion of soluble FAPα protein, an effect that cannot be reversed by αPD‐1/αPD‐L1 treatment alone. Analysis of bone marrow samples from patients with relapsed/refractory multiple myeloma (RRMM) revealed an increased proportion of double‐negative T cells (DNTs), accompanied by reduced granzyme B (GZMB) secretion. Collectively, our findings demonstrate that FAPα^+^ macrophages drive MM immune escape through two distinct mechanisms: PD‐L1 upregulation and T cell senescence acceleration. These results highlight FAPα^+^ macrophages as a promising therapeutic target for overcoming immunotherapy resistance in MM.

## 2 Results

### 2.1 FAPα^+^ Macrophages are Enriched in the Myeloma Microenvironment and Correlate With Disease Progression

**FIGURE 1:**
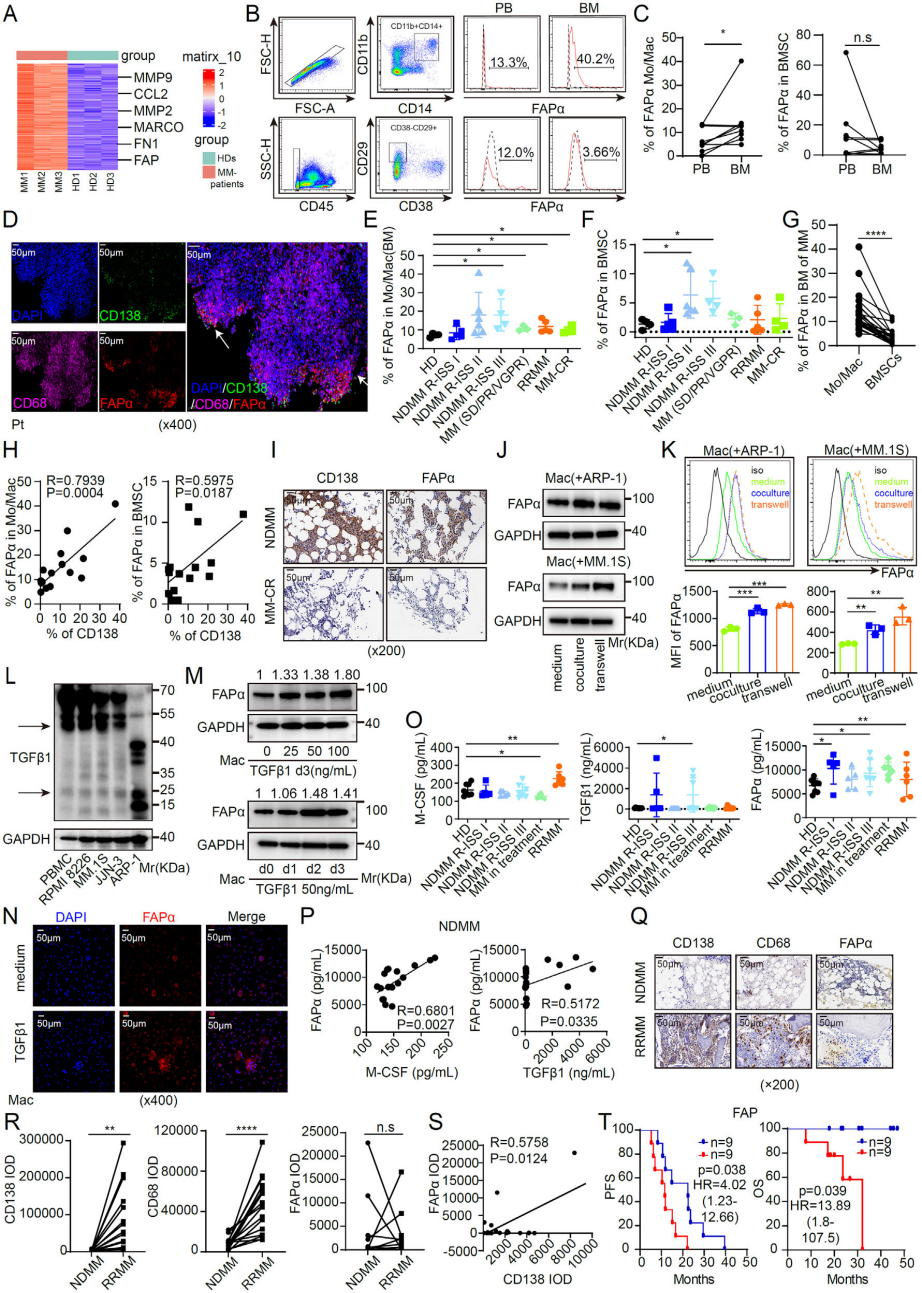
FAPα^+^ Macrophages Are Enriched in the Myeloma Microenvironment and Correlate with Disease Progression. (A) Heatmap of gene expression in macrophages from patients with MM (n = 3) or healthy donors (HDs, n = 3). (B,C) Cell percentages of FAPα^+^ macrophages (CD11b^+^CD14^+^) and FAPα^+^ BMSC (CD45^−^CD38^−^CD29^+^) in PBMCs or BMMCs from patients with MM (n = 9). (D) Reprehensive immunofluorescence staining (IF) images of CD138, CD68, and FAPα in the BM of a NDMM patient (Magnification ×400. Scale bar, 50 µm). (E–G) Flow cytometry analysis of the cell percentages of FAPα^+^ macrophages and FAPα^+^ BMSCs in BMMCs from patients with MM at different disease stages (n = 27) or HDs (n = 4). (H) Correlation analysis of the cell percentages between CD138^+^ MM cells and FAPα^+^ macrophages or FAPα^+^ BMSCs in patients with NDMM (n = 15). (I) Reprehensive immunohistochemical staining (IHC) images of CD138 and FAPα in patients with NDMM or MM‐CR (Magnification ×200. Scale bar, 50 µm). (J,K) Western blot (J) and flow cytometry (K) analysis of FAPα expression in macrophages co‐cultured with MM cells (n = 3). (L) TGFβ1 expression in different MM cell lines. (M‐N) TGFβ1‐induced FAPα protein expression in macrophages (Magnification ×400. Scale bar, 50 µm). (O) M‐CSF, TGFβ1, and FAPα levels in BM supernatants from patients with MM at different disease stages (n = 37) using ELISA assay. (P) Correlation analysis of FAPα and TGFβ1 or M‐CSF in BM supernatant from patients with NDMM (n = 17). (Q) Reprehensive IHC images of bone marrow samples from patients with MM (n = 18) (Magnification ×200. Scale bar, 50 µm). (R) IOD values of CD138, CD68, and FAPα in patients with NDMM or RRMM (n = 18). (S) Correlation analysis between CD138 and FAPα in patients with NDMM. (T) Kaplan–Meier curves of PFS and overall OS in the set of patients with NDMM based on FAP protein expression level detected in tumor tissues. The median value of FAP RNA expression in the was 88.26 (IOD). The expression value of the FAP high group (n = 9) was >88.26(IOD) and the FAP low group (n = 9) was <88.26(IOD). Data are presented as mean ± SD. Each dot means independent samples. ns, no significant difference. ^*^ *p* < 0.05, ^**^ *p* < 0.01, ^***^ *p* < 0.001, ^****^ *p* < 0.0001. Statistical analysis was performed using a 2‐tailed Student's *t*‐test in C, E, F, G, K, O, and R, a Pearson correlation in H, P, and S, a log‐rank test in T. MM, multiple myeloma; HD, healthy donors; BMSCs, bone marrow mesenchymal stem cells; PBMCs, peripheral blood mononuclear cells; BMMCs, bone marrow mononuclear cells; BM, bone marrow; NDMM, newly diagnosed MM; RRMM, relapsed or refractory MM; CR, complete response; TGFβ1, Transforming growth factor beta 1; M‐CSF, macrophage colony stimulating factor; IHC, Immunohistochemistry; IOD, Integrated Optical Density; RRMM, Relapsed/Refractory MM; PFS, Progression‐free survival; OS, overall survival.

Tumor‐associated macrophages (TAMs) are pivotal components of the multiple myeloma (MM) tumor microenvironment (TME), driving chemoresistance, immunosuppression, and metastasis [26, 27]. While classical M1/M2 polarization has dominated prior TAM research, emerging evidence reveals unanticipated TAM heterogeneity, with distinct functional subsets remaining incompletely defined [24]. To identify novel MM‐associated TAM subsets, we isolated macrophages from healthy donors (HDs) and MM patients for RNA sequencing (RNA‐seq). As shown in Figure 1A, *fibroblast activation protein α (FAPα)* was significantly upregulated in macrophages from MM patients versus HDs, along with significant enrichment of some well‐established important genes in MM, such as *CCL2*, *MMP2*, *MMP9*, *MACRO*, and *FN1* [28, 29, 30, 31]. Flow cytometry (FCM) confirmed markedly higher membrane‐bound FAPα expression on CD11b^+^CD14^+^ bone marrow (BM) macrophages compared to peripheral blood mononuclear cells (PBMCs) or BM stromal cells (BMSCs; CD45^−^CD29^+^CD38^−^). These data establish FAPα^+^ macrophages—rather than FAPα^+^ BMSCs—as the predominant FAPα^+^ population specifically enriched in the MM BM niche (Figure 1B,C). Spatially, immunofluorescence (IF) staining localized FAPα^+^ macrophages in proximity to CD138^+^ myeloma cells (Figure 1D), implicating paracrine crosstalk with malignant plasma cells in FAPα induction. Both FAPα^+^ macrophages and FAPα^+^ BMSCs were elevated across MM disease stages, with FAPα^+^ macrophages consistently outnumbering FAPα^+^ BMSCs (Figure 1E–G). FAPα^+^ macrophage frequency positively correlated with CD138^+^ tumor burden in newly diagnosed MM (NDMM) and was barely detectable in MM patients achieving complete response (MM‐CR) (Figure 1H,I), supporting FAPα^+^ macrophages as a potential disease biomarker. To dissect the mechanism of FAPα induction, primary macrophages were treated with conditioned medium (CM) from MM cell lines (ARP‐1, MM.1S). MM cell‐derived CM significantly upregulated FAPα expression (Figure 1J,K). Given that transforming growth factor beta 1 (TGFβ1) induces FAPα in activated fibroblasts [32], we hypothesized that MM‐secreted TGFβ1 mediates FAPα upregulation in macrophages. Consistent with this, recombinant TGFβ1 or TGFβ1‐enriched MM cell CM directly induced FAPα in macrophages (Figure 1L–N). Cytokine profiling of MM BM supernatants revealed elevated TGFβ1 and macrophage colony‐stimulating factor (M‐CSF) in NDMM and relapsed/refractory MM (RRMM) patients, with concentrations positively correlating with FAPα^+^ macrophage frequency (Figure 1O,P). To validate clinical relevance, immunohistochemical (IHC) staining of 18 paired NDMM/RRMM BM sections showed higher CD138 (myeloma marker) and CD68 (macrophage marker) expression in RRMM (Figure 1Q,R). Notably, FAPα positively correlated with CD138 in NDMM (Figure 1S), and high FAPα expression in NDMM was associated with poorer prognosis (Figure 1T). Collectively, these findings identify FAPα^+^ macrophages—induced by MM‐secreted TGFβ1—as the dominant FAPα^+^ population in the MM BM niche. Their frequency correlates with NDMM disease stage and prognosis, highlighting FAPα^+^ macrophages as both a predictive biomarker and promising therapeutic target for MM.

### 2.2 FAPα Promotes PD‐L1 Expression in Macrophages to Mediate T Cell Suppression

Tumor‐associated macrophages (TAMs) drive chemoresistance, immunosuppression, and metastasis in MM [25]. To validate TAM‐mediated immune suppression in MM, we depleted TAMs via intratumoral injection of clodronate liposomes [33] in 5TGM1 tumor‐bearing mice. In vivo, combination therapy with clodronate liposomes and anti‐PD‐1 antibody significantly suppressed tumor growth versus liposome controls (Figure S1A–C). IHC staining of tumor tissues for CD138, F4/80, CD4, and CD8 showed that TAM depletion + anti‐PD‐1 therapy markedly enhanced CD4^+^ and CD8^+^ T cell infiltration (Figure S1D,E), highlighting TAM‐expressed PD‐L1 as a key driver of the immunosuppressive TME.

**FIGURE 2:**
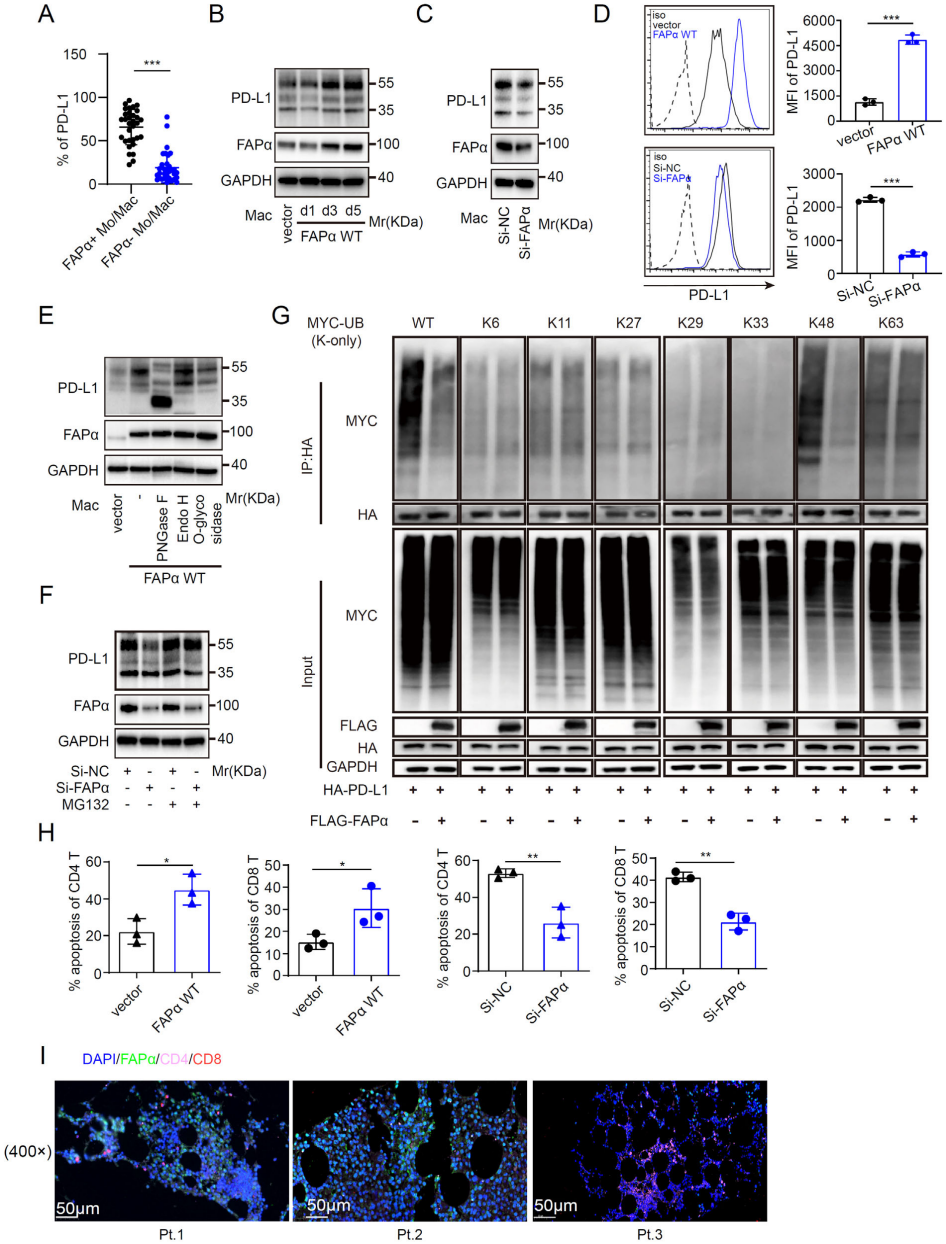
FAPα Promotes PD‐L1 Expression in Macrophages to Mediate T Cell Suppression. (A) Flow cytometry analysis of PD‐L1 expression in FAPα^+^ versus FAPα^−^ macrophages from BMMCs of patients with MM (n = 32). (B–D) WB (B,C) and flow cytometry (D) analysis of PD‐L1 expression in macrophages transfected with control AAV (vector) or FAPα wild‐type AAV (FAPα WT), and control siRNA (Si‐NC) or FAPα‐targeting siRNA (Si‐FAPα) (n = 3). (E) WB analysis of PD‐L1 glycosylation pattern after treatment with PNGase F, Endo H, and O‐glycosidase. (F) WB analysis of PD‐L1 expression in Si‐NC or Si‐FAPα silenced macrophages treated with or without MG‐132. (G) Immunoblot analysis of ubiquitination of HA‐tagged PD‐L1 following co‐immunoprecipitation (CO‐IP) of HA‐PD‐L1 with anti‐HA antibody from lysates of HEK293T cells co‐transfected with indicated plasmids. (H) Flow cytometry analysis of the cell apoptosis in CD4 and CD8 T cells after co‐culture with FAPα WT or Si‐FAPα macrophages (n = 3). (I) IF staining of FAPα, CD4, and CD8 in BM samples from three NDMM patients (Magnification ×400. Scale bar, 50 µm). Data are presented as mean ± SD. Each dot means independent samples. ns, no significant difference. ^*^ *p* < 0.05, ^**^ *p* < 0.01, ^***^ *p* < 0.001. Statistical analysis was performed using a 2‐tailed Student's *t*‐test in A, D, and H. FAPα, fibroblast activation protein alpha; PD‐L1, programmed death‐ligand 1; MM, multiple myeloma; BMMCs, bone marrow mononuclear cells; WB, Western blot; AAV, adeno‐associated virus; siRNA, small interfering RNA; IF, Immunofluorescence; BM, bone marrow; NDMM, newly diagnosed multiple myeloma.

Given the strong correlation between FAPα^+^ macrophages and MM progression, we dissected their role in regulating tumor immunity. FCM revealed substantially higher membrane PD‐L1 expression in FAPα^+^ versus FAPα^−^ macrophages from MM patients (Figure 2A), with functional assays confirming that FAPα positively regulates macrophage PD‐L1 (Figure 2B–D). PD‐L1 stability is tightly controlled by glycosylation, with aberrant glycosylation linked to endoplasmic reticulum (ER) accumulation and ER‐associated protein degradation (ERAD) [34, 35, 36, 37]. We found FAPα promotes PD‐L1 glycosylation in macrophages: treatment with PNGase F (but not Endo H or O‐glycosidase) abrogated FAPα‐enhanced PD‐L1 protein levels (Figure 2E) [36]. Mechanistically, FAPα‐mediated glycosylation impaired PD‐L1 K48‐linked ubiquitination, rendering it resistant to MG‐132‐induced (25 µM, 6 h) proteasomal degradation and stabilizing PD‐L1 expression (Figure 2F,G). Notably, PD‐L1 downregulation upon FAPα knockdown in TAMs was not rescued by the autophagy inhibitor Bafilomycin A1 (100 nM, 12 h), excluding autophagy as a major pathway for FAPα‐mediated PD‐L1 stabilization (Figure S2A). PD‐L1 expression suppress anti‐tumor immunity primarily by inducing T cell apoptosis and exhaustion [38, 39]. Consistent with this, co‐culture assays showed FAPα^+^ macrophages enhanced CD4^+^ and CD8^+^ T cell apoptosis (Figure 2H)—a finding validated by analysis of BM samples from MM patients (Figure 2I). Collectively, these data establish that FAPα promotes macrophage PD‐L1 expression via glycosylation‐dependent stabilization (inhibiting proteasomal degradation), driving T cell apoptosis and MM immune evasion.

### 2.3 FAPα Induces PD‐L1 Expression Through Vimentin S72 Phosphorylation Dependent on FAPα Enzymatic Activity

**FIGURE 3:**
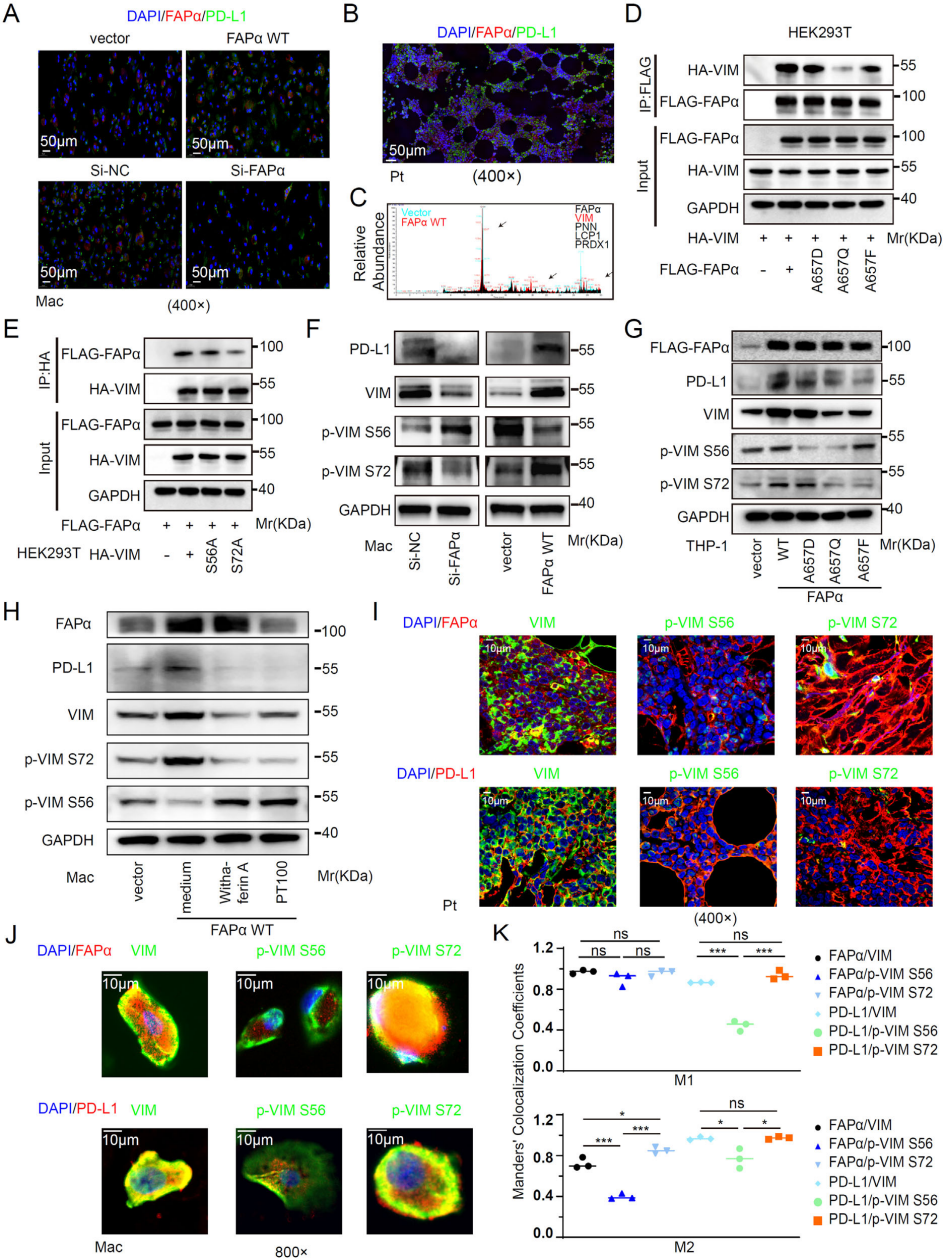
FAPα Induces PD‐L1 Expression Through Vimentin S72 Phosphorylation Dependent on FAPα Enzymatic Activity. (A,B) IF staining of FAPα and PD‐L1 in transfected macrophages (A) and a sample from patient with MM (B) (Magnification ×400. Scale bar, 50 µm). (C) Co‐immunoprecipitation‐mass spectrometry analysis of FAPα‐binding proteins. (D) CO‐IP analysis of VIM binding to different FAPα mutants in HEK293T cells. (E) CO‐IP analysis of the binding ability of FAPα with different VIM mutants in HEK293T cells. (F) WB analysis of PD‐L1, VIM and phospho‐VIM in macrophages transfected with FAPα WT AAV, Si‐FAPα siRNA, or its parallel control. (G) WB analysis of PD‐L1, VIM, and phospho‐VIM in THP‐1 cells overexpressing with FAPα mutant plasmids or its parallel vector. (H) WB analysis of PD‐L1, VIM, and phospho‐VIM expression in FAPα WT‐expressed macrophages treated with the VIM inhibitor withaferin A or the FAPα inhibitor PT100. (I) Co‐localization analysis of FAPα and VIM/p‐VIM, as well as PD‐L1 and VIM/p‐VIM in the tissue from a patient with MM. (Magnification ×400. Scale bar, 10 µm). (J) High‐magnification confocal images of FAPα and p‐VIM S72 colocalization (Magnification ×800. Scale bar, 10 µm). (K) Quantification analysis of FAPα and VIM/p‐VIM colocalization by Manders' colocalization coefficient (MCC). Data are presented as mean ± SD. Each dot means independent samples. ns, no significant difference. ^*^ *p* < 0.05, ^**^ *p* < 0.01, ^***^ *p* < 0.001. Statistical analysis was performed using a 2‐tailed Student's *t*‐test in K. FAPα, fibroblast activation protein alpha; PD‐L1, programmed cell death‐ligand 1; WB, Western blot; VIM, vimentin; CO‐IP, co‐immunoprecipitation; AAV, adeno‐associated virus; siRNA, small interfering RNA; MM, multiple myeloma.

To dissect the mechanism underlying FAPα‐mediated PD‐L1 upregulation, we first tested for direct FAPα‐PD‐L1 interaction. Fluorescence confocal imaging showed no colocalization between FAPα and PD‐L1 in macrophages (Figure 3A,B), ruling out direct binding. Given that FAPα enzymatic activity and substrate binding are mutation‐dependent [40], with Ala657 critical for endopeptidase activity and substrate recognition (Figure S3A), we performed co‐immunoprecipitation (co‐IP) with anti‐FLAG beads in HEK293T cells transfected with wild‐type (WT) FAPα or its Ala657 mutants (A657D, A657Q, A657F), followed by mass spectrometry. Among candidate interactors, vimentin (VIM)—a known PD‐L1 regulator in metastatic lung adenocarcinoma [41] —was prioritized for further study (Figure 3C). Exogenous co‐IP confirmed strong interaction between VIM and WT FAPα, with weaker binding to FAPα mutants (A657Q/A657F > A657D, relative to WT; Figure 3D). Reciprocal co‐IP validated that FAPα binds robustly to WT VIM but weakly to the S72A phosphorylation‐deficient mutant (Figure 3E), implicating critical function of VIM S72 phosphorylation in FAPα‐VIM complex formation. In macrophages, FAPα positively regulated total VIM, VIM S72 phosphorylation, and downstream PD‐L1, while negatively regulating VIM S56 phosphorylation (Figure 3F)—consistent with reports that VIM S56 phosphorylation promotes VIM degradation [42, 43]. In THP‐1‐derived macrophages, WT FAPα increased total VIM and VIM^S72^ phosphorylation relative to controls. In contrast, loss of FAPα enzymatic activity (via Ala657 mutation) reduced VIM^S72^ phosphorylation but elevated VIM^S56^ phosphorylation (Figure 3G), confirming that FAPα enzymatic activity is required for VIM^S72^ phosphorylation and FAPα‐VIM interaction. Treatment of macrophages with the VIM inhibitor Withaferin‐A or FAPα inhibitor PT100 (Talabostat), a potent DPPIV Activity Structure Homolog family serine protease inhibitor that targets FAP's catalytic site [44], impaired VIM^S72^ phosphorylation and PD‐L1 expression, while increasing VIM^S56^ phosphorylation (Figure 3H). Fluorescence confocal imaging in macrophages and MM patient‐derived extramedullary tissues confirmed colocalization of FAPα/PD‐L1 with VIM and phosphorylated VIM^S72^ (Figure 3I–K). Previous studies show VIM induces PD‐L1 nuclear translocation, where nuclear PD‐L1 interacts with transcription factors (e.g., STAT3, p65, c‐Jun) to amplify PD‐L1 gene expression [45, 46]. Consistently, similar results were detected in macrophages (Figure S3B,C). Collectively, these data establish that FAPα upregulates PD‐L1 expression in macrophages via VIM^S72^ phosphorylation—mediated by FAPα enzymatic activity—coupled with VIM‐dependent PD‐L1 transcriptional amplification.

### 2.4 FAPα^+^ Macrophages Accelerate T Cell Senescence and Impair Anti‐Tumor T Cell Function

**FIGURE 4:**
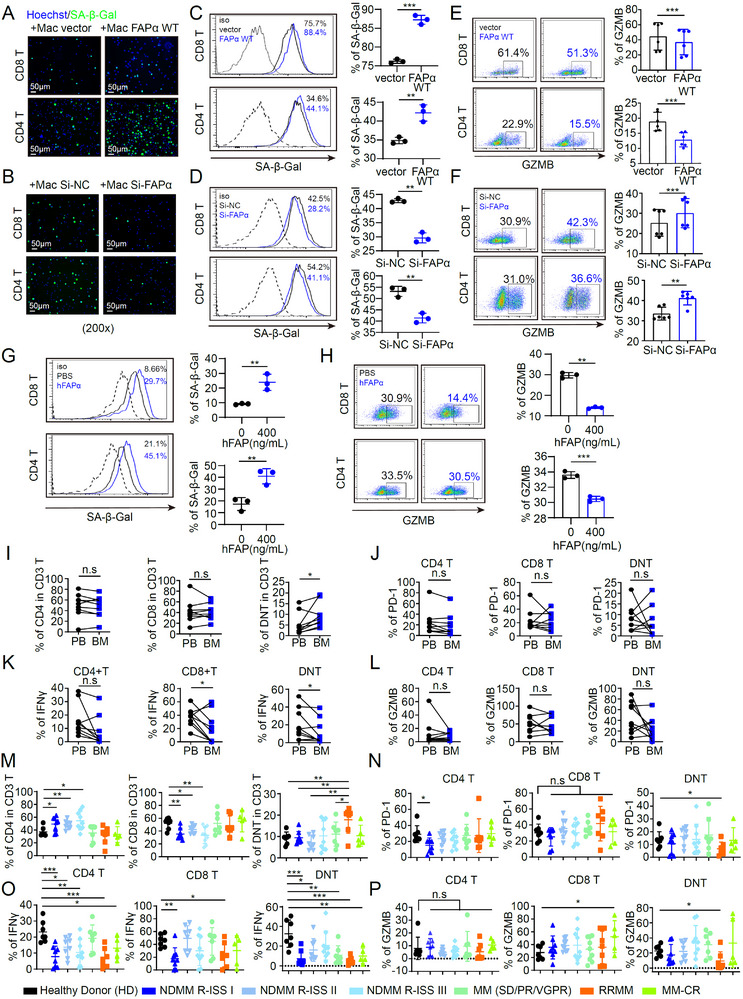
FAPα^+^ Macrophages Accelerate T Cell Senescence and Impair Anti‐Tumor T Cell Function. (A–D) IF (A,B) and flow cytometry (C,D) analysis of SA‐β‐gal expression in CD8 T and CD4 T cells co‐cultured with FAPα WT (n = 3) or Si‐FAPα (n = 3) macrophages (Magnification ×200. Scale bar, 50 µm). (E,F) Flow cytometry analysis of GZMB expression in CD8 T and CD4 T cells co‐cultured with FAPα WT (n = 6) or Si‐FAPα (n = 6) macrophages. (G,H) Flow cytometry analysis of SA‐β‐Gal (G) and GZMB (H) expression in CD8 T and CD4 T cells treated with recombinant human FAPα. (I–L) Flow cytometry analysis of CD4 T, CD8 T, DNT cells (I), and expression of PD‐1 (J), IFNγ (K) and GZMB (L) in PBMCs and BMMCs from patients with MM (n = 9). (M‐P) Flow cytometry analysis of immune cell subsets and effector molecules in patients with MM at different disease stages (n = 51), including cell percentages of CD4 T, CD8 T, and DNT cells among CD3^+^T cells (M), and PD‐1 (N), IFNγ (O), and GZMB (P) expression in these subsets. Data are presented as mean ± SD. Each dot means independent samples. ns, no significant difference. ^*^ *p* < 0.05, ^**^ *p* < 0.01, ^***^ *p* < 0.001. Statistical analysis was performed using a 2‐tailed Student's *t*‐test in C‐P. FAPα, fibroblast activation protein alpha; SA‐β‐Gal, senescence‐associated β‐galactosidase; GZMB, granzyme B; DNT, double negative T cells; PD‐1, programmed cell death protein‐1; IFNγ, Interferon gamma; PBMCs, peripheral blood mononuclear cells; BMMCs, bone marrow mononuclear cells; MM, multiple myeloma; BM, bone marrow; IF, Immunofluorescence.

T cell senescence is a key driver of tumor immune escape, characterized by impaired antigen responsiveness, proliferation arrest, and reduced cytotoxic capacity [47, 48]. Using senescence‐associated β‐galactosidase (SA‐β‐Gal)—a canonical senescence biomarker—we found that FAPα^+^ macrophages promoted senescence in both CD4^+^ and CD8^+^ T cells (Figure 4A–D). Consistent with reports that senescent T cells exhibit defective cytokine secretion [49]. FAPα^+^ macrophages induced T cell senescence alongside reduced granzyme B (GZMB) production (Figure 4E, F). Given that FAPα is secreted by multiple cell types [50, 51], we tested recombinant human FAPα (hFAP) to dissect its direct role. Notably, hFAP directly induced T cell senescence, leading to decreased GZMB and CD28 expression in CD4^+^/CD8^+^ T cells (Figure 4G,H; Figure S4A) without affecting T cell apoptosis (Figure S4B). These data indicate FAPα^+^ macrophages impair T cell function via secreted FAPα‐mediated senescence—independent of the PD‐1/PD‐L1 axis.

To validate clinical relevance, we analyzed paired peripheral blood (PB) and BM samples from 9 MM patients. Unexpectedly, double‐negative T cells (DNTs)—a subset with anti‐tumor potential [52, 53, 54]—were enriched in BM versus PB, with reduced interferon gamma (IFNγ) secretion (Figure 4I–L), suggesting BM‐resident FAPα^+^ macrophages remodel the TME by inhibiting DNT function. Immune profiling of MM patient BM across disease stages revealed: (i) increased CD4^+^ T cells and reduced CD8^+^ T cells in NDMM; (ii) elevated DNTs in RRMM; (iii) decreased IFNγ production in CD4^+^/CD8^+^ T cells in NDMM/RRMM, with higher GZMB^+^CD8^+^ T cells in MM‐CR; (iv) low PD‐1‐expressing DNTs in RRMM showed reduced IFNγ and GZMB (Figure 4M–P). Subset analysis revealed unchanged Th2/Tc2/Th22/Tc22 populations, a trend toward increased Th17/Tc17 in some NDMM patients (Figure S4C,D), significantly elevated IL‐9^+^/IL‐10^+^ CD4^+^ T cells, and impaired perforin^+^ CD8^+^ T cells in RRMM (Figure S4E,F). Notably, CD4^+^ Treg frequency was unaltered in the MM patient BM (Figure S4G). In vitro assays confirmed hFAP inhibited IL‐2, IFNγ, and GZMB expression in DNTs (Figure S4H,I). As hFAPα suppressed both CD4^+^T and CD8^+^T cell function, we checked the common key signaling protein, CD3ε, and its phosphorylation, p‐Tyr‐CD3ε.As expected, hFAPα significantly inhibited phosphorylation of p‐CD3εTyr of the T cell line (Jurkat), which may result in impaired signaling and T cell function (Figure S4J). Collectively, these findings establish that FAPα^+^ macrophages secrete FAPα to accelerate T cell senescence and impair the function of anti‐tumor T cell subsets (CD4^+^/CD8^+^ T cells, DNTs), further reinforcing the immunosuppressive TME in MM.

### 2.5 FAPα Inhibition Alleviates Anti‐PD‐1/PD‐L1 Therapy Resistance In Vitro

**FIGURE 5:**
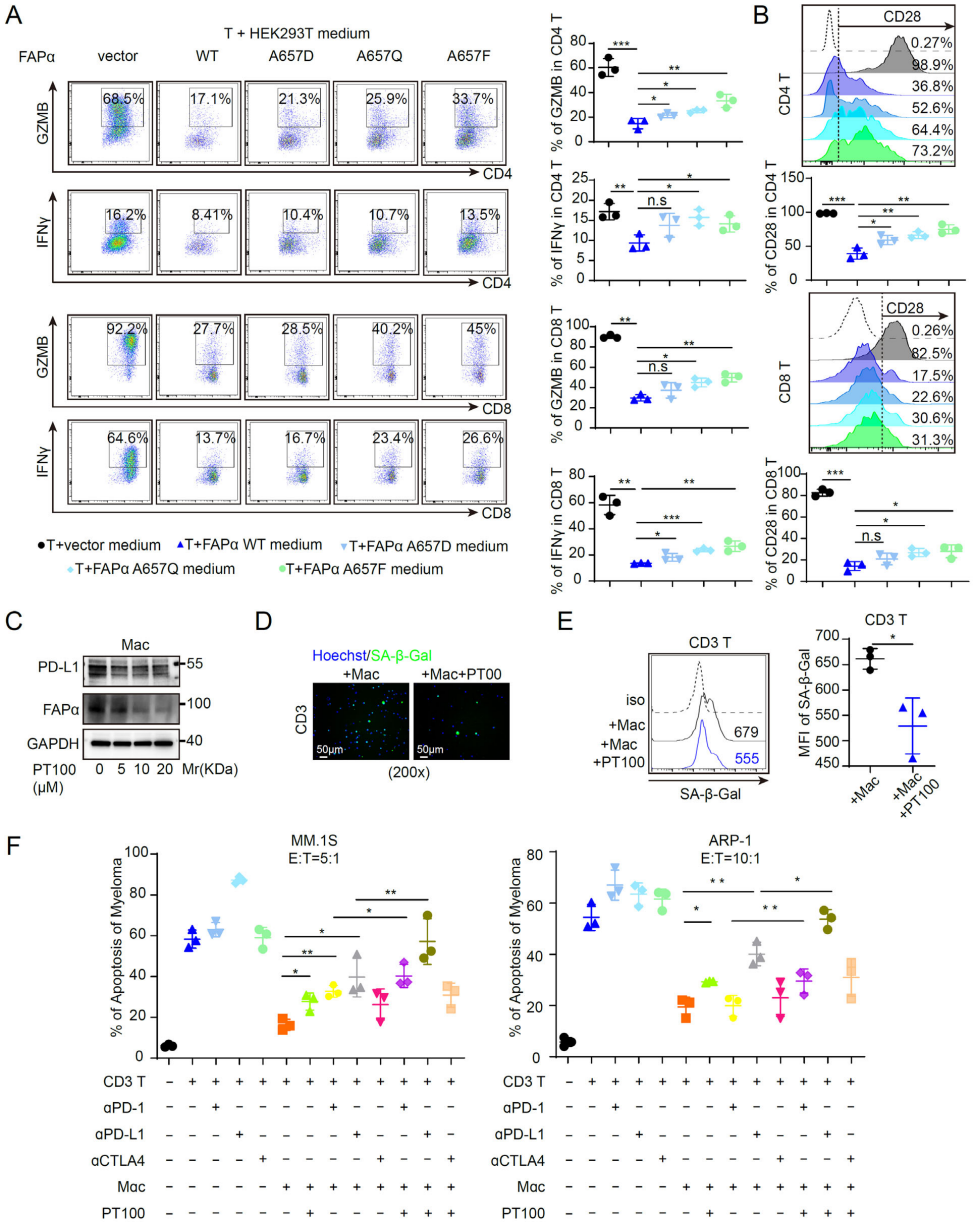
FAPα Inhibition Alleviates Anti‐PD‐1/PD‐L1 Therapy Resistance In Vitro. (A,B) IFNγ, GZMB (A), and CD28 (B) expression on CD4 T and CD8 T cells treated with supernatant from HEK293T cells expressing FAPα WT or its mutants. (C) WB analysis of FAPα and PD‐L1 expression in macrophages treated with the FAPα inhibitor PT100. (D‐E) IF (D) and flow cytometry (E) analysis of SA‐β‐Gal expression in CD3^+^ T cells co‐cultured with PT100‐treated macrophages (Magnification ×200. Scale bar, 50 µm). (F) Analysis of MM cell apoptosis co‐cultured with different immune cell combinations (CD3^+^ T cells and/or macrophages) under different treatment conditions (FAPα inhibitor, αPD‐L1, αPD‐1, or αCTLA‐4 antibodies, alone or in combination) as indicated. Data are presented as mean ± SD. Each dot means independent samples. ns, no significant difference. ^*^ *p* < 0.05, ^**^ *p* < 0.01, ^***^ *p* < 0.001. Statistical analysis was performed using a 2‐tailed Student's *t*‐test in A, B, E, and F. FAPα, fibroblast activation protein alpha; IFNγ, Interferon gamma; GZMB, granzyme B; WB, Western blot; PD‐L1, programmed death‐ligand 1; SA‐β‐Gal, senescence‐associated β‐galactosidase; MM, multiple myeloma; PD‐1, programmed cell death protein‐1; CTLA4, cytotoxic T‐lymphocyte‐associated protein 4.

To confirm FAPα’s direct role in T cell dysfunction, we treated activated T cells with CM from HEK293T cells lenti‐virally transduced with WT FAPα or enzymatically inactive FAPα mutants. CM from WT FAPα‐expressing cells significantly suppressed GZMB, IFNγ, and CD28 expression in CD4^+^/CD8^+^ T cells, whereas loss of FAPα enzymatic activity partially restored T cell function (Figure 5A, B)—consistent with FAPα’s serine protease‐dependent mechanism [40]. This immunosuppressive effect was recapitulated with CM from HS‐5 cells (a human BM stromal cell line) and was not reversed by αPD‐1/αPD‐L1 blockade (Figure S5A–D), confirming PD‐1/PD‐L1 independence of FAPα‐mediated T cell suppression. from the Treatment of macrophages with the FAPα inhibitor PT100 reduced macrophage PD‐L1 expression (Figure 5C). Notably, T cells cocultured with PT100‐pretreated macrophages showed decreased senescence‐associated β‐galactosidase (SA‐β‐Gal) activity (Figure 5D,E), demonstrating that FAPα inhibition abrogates macrophage‐induced T cell senescence. Consistent with TAM‐mediated impairment of anti‐tumor immunity in MM [55], TAMs significantly reduced T cell cytotoxicity against MM cells. Critically, combination treatment with PT100 and αPD‐1/αPD‐L1 antibodies synergistically enhanced anti‐tumor efficacy in vitro, whereas PT100 failed to augment αCTLA‐4 therapy (Figure 5F). This aligns with clinical observations that CTLA‐4 blockade exhibits limited efficacy in MM due to stage‐specific checkpoint dysregulation [56], further supporting FAPα‐PD‐1/PD‐L1 crosstalk as a key therapeutic axis. Collectively, these data establish that FAPα in TAMs drives T cell senescence and dysfunction via its enzymatic activity, promoting resistance to αPD‐1/αPD‐L1 therapy. FAPα inhibition with PT100 reverses T cell senescence, reduces macrophage PD‐L1 expression, and synergizes with αPD‐1/αPD‐L1 blockade—providing a rationale for targeting FAPα to overcome immunotherapy resistance in MM.​

### 2.6 FAPα Inhibition Overcomes Immunotherapy Resistance in MM by Depleting TAMs and Enhancing GZMB‐Mediated Cytotoxicity

**FIGURE 6:**
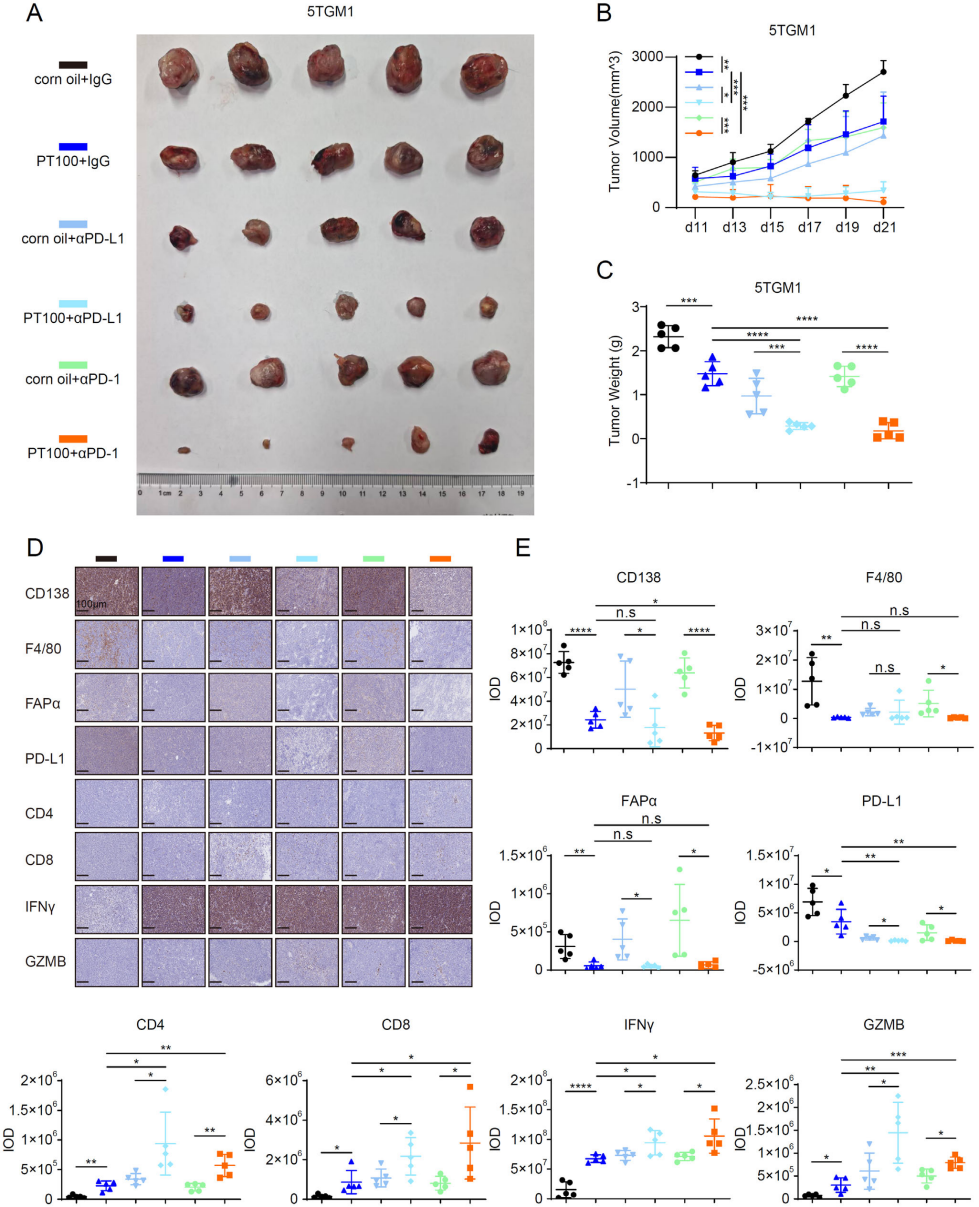
FAPα Inhibition Overcomes Immunotherapy Resistance in MM by Depleting TAMs and Enhancing GZMB‐Mediated Cytotoxicity. (A–C) Representative images of 5TGM1 tumors (A), tumor growth curves (B), and tumor weight (C) from the indicated treatment groups. (D‐E) IHC staining (D) and corresponding IOD analysis (E) of CD138, F4/80, FAPα, PD‐L1, CD4, CD8, IFNγ, and GZMB in 5TGM1 tumors (Magnification ×200. Scale bar, 100 µm). Data are presented as mean ± SD. Each dot means independent samples. ns, no significant difference. ^*^ *p* < 0.05, ^**^ *p* < 0.01, ^***^ *p* < 0.001, ^****^ *p* < 0.0001. Statistical analysis was performed using a 2‐way ANOVA test in B, a 2‐tailed Student's *t*‐test in C and E. FAPα, fibroblast activation protein alpha; PD‐L1, programmed cell death‐ligand 1; PD‐1, programmed cell death protein‐1; IHC, Immunohistochemistry; IOD, Integrated Optical Density; IFNγ, Interferon gamma; GZMB, granzyme B.

To validate the therapeutic potential of targeting FAPα in TAMs, we evaluated the FAPα inhibitor PT100 alone or in combination with αPD‐1/αPD‐L1 antibodies in the 5TGM1 syngeneic MM mouse model—a well‐established system for recapitulating human MM bone marrow tropism and immune evasion [57]. Notably, PT100 monotherapy significantly suppressed tumor growth in vivo, consistent with its reported T cell‐independent anti‐tumor activity via innate immune activation [58]. Critically, combination treatment with PT100 and αPD‐1/αPD‐L1 antibodies exerted superior anti‐tumor efficacy compared to either monotherapy, as evidenced by reduced tumor burden (Figure 6A–C). Consistently, IHC staining confirmed that combination therapy diminished intratumoral expression of FAPα, F4/80, and PD‐L1—while concomitantly enhancing CD4^+^/CD8^+^ T cell infiltration and GZMB secretion (Figure 6D; Figure S6A). Collectively, these in vivo data establish that PT100 targets FAPα to deplete TAMs, reduce TAM‐derived PD‐L1 expression, and augment αPD‐1/αPD‐L1‐mediated anti‐tumor immunity via enhanced GZMB secretion. This dual mechanism—TAM depletion and restored cytotoxic T cell function—overcomes immunotherapy resistance in MM, reinforcing PT100 as a promising combinatorial partner for αPD‐1/αPD‐L1‐based regimens.

### 2.7 FAPα Inhibition Potentiates Anti‐PD‐1/PD‐L1 Therapy across Multiple Tumor Types

**FIGURE 7:**
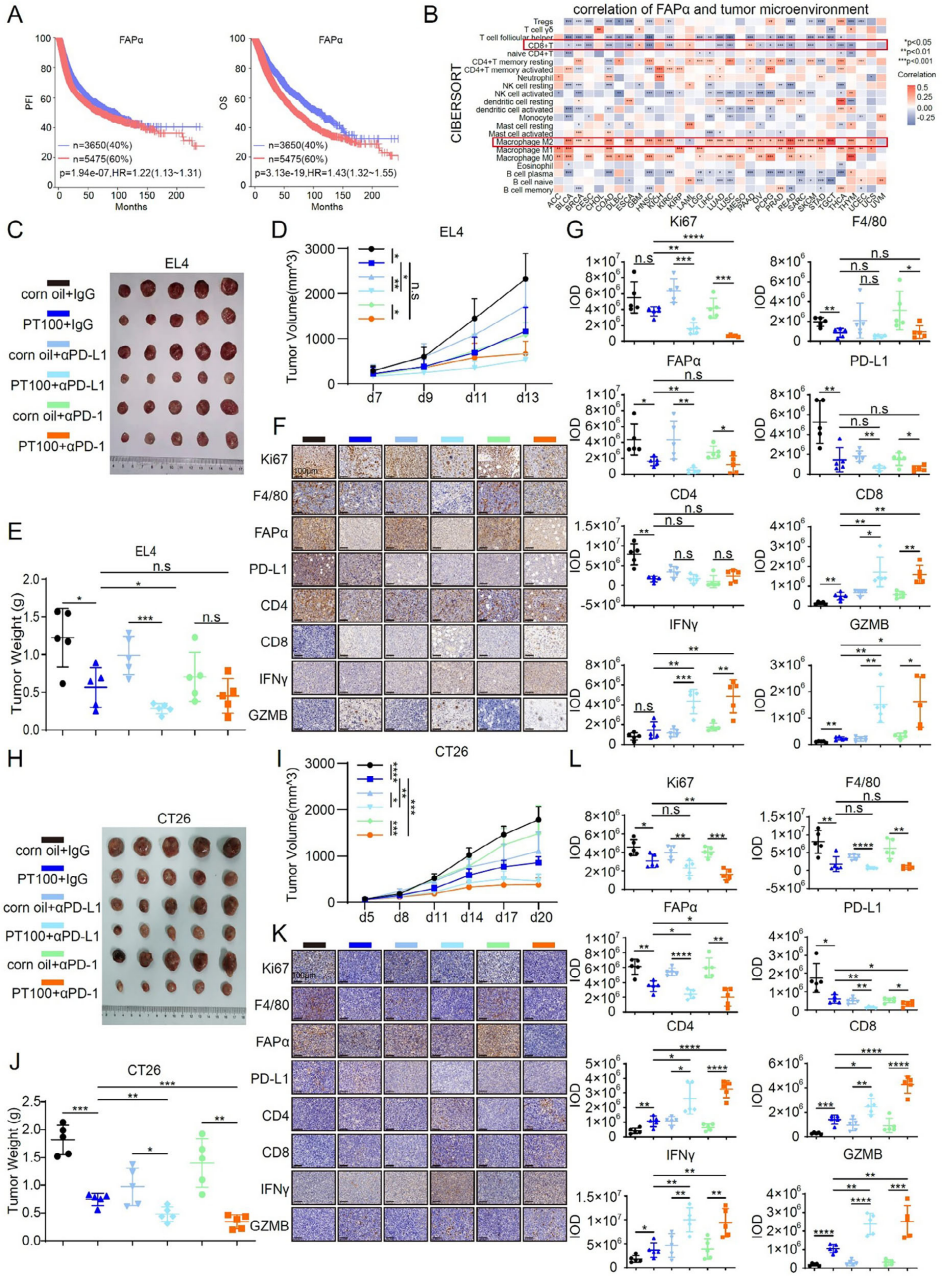
FAPα Inhibition Potentiates Anti‐PD‐1/PD‐L1 Therapy Across Multiple Tumor Types. (A) Correlation analysis of FAPα mRNA expression and PFI / OS in pan‐cancer data. (B) Correlation analysis of FAPα expression and immune cell infiltration in the tumor microenvironment.  (C–E) Representative images(C), tumor growth curves(D), and tumor weight (F) of EL4 tumors from the indicated groups treated by αPD‐1/αPD‐L1 antibodies combined with or without FAPα inhibitor. (F,G) Representative images (F) and IOD analysis of IHC staining (G) of Ki67, F4/80, FAPα, PD‐L1, CD4, CD8, IFNγ, and GZMB in EL4 tumors (Magnification ×200. Scale bar, 100 µm). (H–J) Representative images(H), tumor growth curves(I), and tumor weight (J) of CT26 tumors from the indicated groups treated by αPD‐1/αPD‐L1 antibodies combined with or without FAPα inhibitor. (K, L) Representative images (K) and IOD analysis of IHC staining (L) of Ki67, F4/80, FAPα, PD‐L1, CD4, CD8, IFNγ, and GZMB in CT26 tumors (Magnification ×200. Scale bar, 100 µm). Data are presented as mean ± SD. Each dot means independent samples. ns, no significant difference. ^*^ *p* < 0.05, ^**^ *p* < 0.01, ^***^ *p* < 0.001, ^****^ *p* < 0.0001. Statistical analysis was performed using log‐rank test in A, a 2‐way ANOVA test in D and I,2‐tailed Student's *t*‐test in E, G, J, and L. FAPα, fibroblast activation protein alpha; PFI, progression‐free interval; OS, overall survival; IHC, Immunohistochemistry; IOD, Integrated Optical Density; PD‐L1, programmed cell death‐ligand 1; PD‐1, programmed cell death protein‐1; IFNγ, Interferon gamma; GZMB, granzyme B.

Given the promising results in MM, we explored the broader therapeutic potential of FAPα targeting by extending our studies to other tumor models. TCGA pan‐cancer analysis confirmed that high FAPα expression correlates with poor overall survival across diverse malignancies, including colorectal cancer and lymphoma (Figure 7A)—consistent with FAPα’s role as an independent prognostic risk factor in solid tumors. Immune infiltration analysis further revealed that FAPα expression positively correlates with M2‐polarized macrophages (pro‐tumorigenic subtype) and negatively correlates with CD8^+^ T cells and natural killer (NK) cells across cancer types (Figure 7B), indicating a conserved FAPα‐mediated immunosuppressive TME signature beyond MM. To validate this pan‐cancer potential, we evaluated PT100 alone or in combination with αPD‐1/αPD‐L1 antibodies in syngeneic EL4 T‐lymphoma and CT26 colorectal cancer models—well‐established systems for studying immunotherapy responsiveness. Notably, PT100 monotherapy significantly suppressed tumor growth in both models (Figure 7C–L), mirroring its T cell‐independent anti‐tumor activity observed in MM. Critically, combination treatment with PT100 and αPD‐1/αPD‐L1 antibodies exerted superior tumor suppression compared to either monotherapy, as evidenced by reduced tumor burden (Figure 7C–L). IHC staining confirmed conserved mechanisms across tumor types: combination therapy diminished intratumoral FAPα, F4/80, and PD‐L1 expression—while concomitantly enhancing CD4^+^/CD8^+^ T cell infiltration and GZMB secretion (Figure 7F,G,K,L; Figure S6A,B), underscoring that FAPα inhibition restores anti‐tumor immunity via conserved TME remodeling. Collectively, these data establish that FAPα^+^ TAMs drive immunosuppression in EL4 lymphoma and CT26 colorectal cancer—mirroring their role in MM. FAPα inhibition with PT100 depletes TAMs, reduces PD‐L1 expression, and synergizes with αPD‐1/αPD‐L1 blockade across multiple tumor types. This pan‐cancer efficacy highlights FAPα as a conserved therapeutic target for overcoming immunotherapy resistance, providing a rationale for extending FAPα‐targeted combination regimens to diverse malignancies.

## 3 Discussion

FAPα is a well‐characterized biomarker of CAFs in solid TMEs, where it orchestrates tumor cell survival, proliferation, invasion, and chemoresistance—establishing FAPα as a top‐tier therapeutic target for solid malignancies [59, 60]. However, the functional role of FAPα in immune cells within the TME of hematologic malignancies, particularly multiple MM, remains largely unexplored. Our previous study has demonstrated that FAPα is highly expressed in BMSCs from MM patients, where it promotes MM cell proliferation and confers protection against chemotherapy‐induced apoptosis [61]. Additionally, our series of studies has delineated the phenotypic and functional features of myeloma‐associated macrophages (mMΦs) and their contributions to tumor progression, drug resistance, and immune evasion [22, 62, 63]. Based on these foundations, the current study reveals a previously unrecognized paradigm: FAPα^+^ macrophages, rather than FAPα^+^ BMSCs, represent the predominant FAPα^+^ population in the MM bone marrow niche, with their abundance correlating with disease stage. Mechanistically, FAPα regulates PD‐L1 expression in TAMs to suppress T cell function, while in vivo validation confirms that FAPα inhibition reduces TAM‐derived PD‐L1 and potentiates anti‐PD‐1/PD‐L1 immunotherapy efficacy in MM.

TAMs are critical components of the immunosuppressive TME, with PD‐L1 expression serving as a key mediator of tumor immune escape [64]. Our findings identify FAPα^+^ macrophages—potentially induced by MM cell‐secreted transforming TGFβ1—as a negative prognostic factor in MM. Functional characterization further demonstrates that FAPα^+^ macrophages exhibit elevated PD‐L1 expression, with FAPα stabilizing PD‐L1 protein via enhanced N‐glycosylation, which suppresses K48‐linked ubiquitination and proteasomal degradation. This post‐translational regulation ultimately promotes T cell apoptosis, reinforcing MM immune evasion. Notably, fluorescence confocal imaging failed to detect colocalization between FAPα and PD‐L1 in MM TAMs, prompting further investigation into upstream regulatory mechanisms via Co‐IP/MS. This unbiased approach identified VIM—a canonical CAF/myofibroblast biomarker [65], with reported PD‐L1‐regulatory functions in metastatic lung adenocarcinoma [41] —as a critical FAPα interactor. To our knowledge, this study is the first to report a physical and functional interaction between FAPα and VIM. Mechanistically, we demonstrate that FAPα binds VIM in a manner dependent on FAPα’s Ala657 enzymatic activity and VIM^S72^ phosphorylation. In macrophages, FAPα promotes VIM^S72^ phosphorylation while inhibiting VIM^S56^ phosphorylation—consistent with reports that VIM^S56^ phosphorylation accelerates VIM degradation [45, 46]. This dual regulation of VIM phosphorylation facilitates PD‐L1 nuclear translocation, where nuclear PD‐L1 interacts with transcription factors (e.g., NF‐κB, JAK/STAT) to amplify PD‐L1 gene expression—a finding supported by Co‐IP, signaling, and immunofluorescence analysis. However, critical knowledge gaps remain: the precise biochemical basis by which FAPα Ala657 mutants impair VIM binding, and the direct impact of FAPα’s dipeptidyl peptidase/endopeptidase activity on VIM function and T cell biology, warrant further investigation using targeted biochemical approaches.

Beyond PD‐L1‐mediated T cell suppression, our study uncovers a second, PD‐1/PD‐L1‐independent mechanism by which FAPα^+^ macrophages drive immune evasion: induction of T cell senescence, particularly in PD‐1^low^ double‐negative T cells (DNTs). Ghobrial et al. recently reported conserved immune alterations between BM and PB in smoldering MM [66], with no significant differences in CD4^+^/CD8^+^ T cell counts or PD‐1 expression. In contrast, our analysis of paired BM/PB samples from MM patients reveals BM‐enriched DNTs with reduced IFNγ secretion. DNTs—an anti‐tumor T cell subset developed by Zhang et al. [67, 68, 69] express low PD‐1, suggesting resistance to PD‐1/PD‐L1 blockade. Combined with our observation that FAPα^+^ macrophages accelerate T cell senescence, we propose a model wherein: (i) PD‐1^high^ CD4^+^/CD8^+^ T cells are suppressed via the PD‐1/PD‐L1 axis; and (ii) PD‐1^low^ T cells (including DNTs) are restrained by FAPα‐mediated senescence. Though the molecular underpinnings of this FAPα‐driven process require further dissection.

ICIs targeting PD‐1/PD‐L1 have transformed solid tumor therapy but exhibit limited efficacy in hematologic malignancies [56]. Our study provides a mechanistic explanation for this resistance: TAM depletion via clodronate liposomes potentiates anti‐PD‐1/PD‐L1 therapy in MM, highlighting TAMs as key mediators of ICI resistance in the MM TME. Given the abundance of FAPα^+^ macrophages and their high PD‐L1 expression in MM BM, we evaluated the FAPα inhibitor PT100 in a syngeneic MM mouse model. PT100 monotherapy exerted partial anti‐tumor effects, while combination with anti‐PD‐1/PD‐L1 antibodies achieved superior efficacy—demonstrating that targeting FAPα^+^ macrophages reverses immune resistance and restores ICI sensitivity in MM. Extending these findings, pan‐cancer analysis of TCGA data revealed that FAPα expression correlates positively with M2 macrophage infiltration and negatively with overall survival across diverse malignancies, suggesting conserved FAPα^+^ macrophage‐mediated immunosuppression beyond MM. Subsequent validation in syngeneic EL4 T‐lymphoma and CT26 colorectal cancer models confirmed that PT100 + anti‐PD‐1/PD‐L1 combination therapy exhibits pan‐cancer efficacy, supporting the broad translational potential of this regimen.

Despite these advances, our study has limitations. First, the molecular mechanism by which secreted FAPα accelerates T cell senescence remains poorly defined and requires further investigation. Second, in vivo experiments focused on short‐term tumor growth inhibition, and long‐term studies are needed to evaluate tumor recurrence and adaptive resistance—including whether FAPα^+^ macrophages upregulate alternative immune checkpoints (e.g., TIM‐3, LAG‐3) following FAPα inhibition. Third, the clinical relevance of FAPα^+^ macrophages as a predictive biomarker requires validation in larger, prospective cohorts.

In conclusion, our findings establish FAPα^+^ macrophages as critical mediators of immune escape in MM via two complementary mechanisms: PD‐L1 upregulation (via VIM^S72^ phosphorylation and N‐glycosylation‐dependent stabilization) and T cell senescence. These results position FAPα^+^ macrophages as both a prognostic biomarker and therapeutic target in MM, while pan‐cancer validation extends this paradigm to other malignancies (Figure S8). The combination of FAPα inhibitors and anti‐PD‐1/PD‐L1 antibodies represents a promising strategy to overcome ICI resistance, offering new hope for patients with MM and other FAPα‐high tumors.

## 4 Experimental Section

### 4.1 Sex as a Biological Variable

Our study utilized both male and female biopsies from humans and mice for the study, as sex was not considered a biological variable.

### 4.2 In Vivo Experimental Therapy in Mouse Models

C57BL/KalwRij mice were provided by Professor Qing Yi. Female C57BL/6 mice and female BALB/c mice (aged 6–8 weeks) were purchased from GemPharmatech Co., Ltd (Nanjing China). All mice were housed in the animal facility of Zhejiang University School of Medicine. Totally 1×10^6 5TGM1 cells were subcutaneously inoculated in each C57BL/KalwRij mouse. Totally 2×10^6 EL4 cells were subcutaneously inoculated in each C57BL/6 mouse. Totally 5×10^5 CT26 cells were subcutaneously inoculated in each BALB/c mouse. After the tumors were visible, the animals were randomly assigned to different groups. The FAPα inhibitor PT100 was administered at 50 µg per mouse per day, and αPD‐L1 or αPD‐1 antibody was administered at 150 µg per mouse, every two or three days. In the macrophage‐depletion 5TGM1 mouse model, clodronate liposome was administered at 200 µl once per mouse by intratumor injection. Tumor diameters were monitored, and tumor volume was calculated as length x width^2^ divided by 2.

### 4.3 Cell Culture and Virus Transfection

RPMI 8226 (RRID: CVCL_0014), MM.1S (RRID: CVCL_8792), JJN‐3 (RRID: CVCL_2078), ARP‐1 (RRID: CVCL_D523), 5TGM1 (RRID: CVCL_VI66), THP‐1 (RRID: CVCL_0006), CT26 (RRID: CVCL_7254) and EL4 (RRID: CVCL_0255) cells were cultured in RPMI 1640 medium. HEK293T (RRID: CVCL_0063) and HS‐5 (RRID: CVCL_3720) cells were cultured in DMEM. All media contained 10% FBS. PBMCs were acquired from healthy donors and MM patients and adherently cultured in DMEM for 1 month to obtain BMSCs. PBMCs were acquired from healthy donors and adherently cultured in DMEM with 25 ng/mL M‐CSF for 7–10 days to obtain macrophages. Recombinant human TGFβ1 was obtained from BioLegend; hFAPα was obtained from R&D Systems. Macrophages and BMSCs were transfected with AAV or normal control virus (VH848275, Vigene Biosciences, China) to induce FAPα overexpression. FAPα siRNA was also obtained from Vigene Biosciences. Transfection of siRNAs was performed with Lipofectamine RNAi MAX (Thermo Fisher Scientific, USA). Green fluorescent protein (GFP), FLAG, and anti‐puromycin‐FLAG‐tagged lentiviruses with different FAPα point mutations and control lentiviruses were obtained from GeneChem. HS‐5 and THP‐1 were transfected with the above lentiviruses for 72 h and selected with puromycin (10 µg/mL) for 3 days. HEK293T cells were transfected with these lentiviruses for 72 h and selected with puromycin (10 µg/mL) for one week.

### 4.4 Western Blotting (WB), Ubiquitination Assays and PD‐L1 Glycosylation Analysis

Western Blotting (WB) analysis was performed as described previously [70]. To detect PD‐L1 ubiquitination, the ubiquitination assay was carried out as described previously. To detect PD‐L1 glycosylation, cell lysates were treated with PNGase F (R&D Systems, USA), Endo H (R&D Systems, USA), and O‐glycosidase (Rhino Bio, USA) as described by the manufacturers and then analyzed by WB.

### 4.5 RNA‐seq and Bioinformatic Analyses

Total RNA was extracted from macrophages transfected with control siRNA or siRNA against FAPα (Si‐FAPα), CD4^+^ T cells, and CD8^+^ T cells activated by beads and treated with or without hFAP. Data were analyzed by the Novogene Bioinformatics Institute. Heatmap analysis was performed with a web tool (http://magic.novogene.com).

### 4.6 Immunofluorescence (IF) and Fluorescence Confocal Analysis

Bone marrow biopsy tissue samples from MM patients, extramedullary tumor tissue samples from MM patients, macrophages cultured from PBMCs, macrophages transfected with control AAV or FAPα WT, and macrophages transfected with control siRNA or Si‐FAPα were fixed with paraformaldehyde and permeabilized with Triton X‐100 for IF staining and fluorescence confocal microscopy. Immuno‐stained cells were examined under a fluorescence microscope (Olympus IX71, Tokyo, Japan).

### 4.7 Co‐Immunoprecipitation

After infection with plasmid for 48 h, cells were lysed in a lysis buffer on ice. Lysates were centrifuged at 12000 g for 10 min, and supernatants were incubated with FLAG‐beads or HA‐beads for 2 h at 4°C. The beads were washed with washing buffer 5 times and boiled with 1x loading buffer for 20 min at 100°C. The beads were isolated, and proteins in supernatants were analyzed by western blotting or mass spectrometry.

### 4.8 Isolation of Human CD4^+^, CD8^+^ and CD3^+^ T Cells and Co‐Culture Experiments

CD4^+^ T cells were isolated from healthy donor PBMCs with the EasySep Human CD4^+^ T Cell Isolation Kit (Cat #17952, STEMCELL Technologies, Canada). CD8^+^ T (Cat #17953) and CD3^+^ T (Cat #17851) cells were isolated with similar kits. All isolation procedures were performed as described by the manufacturer. CD4^+^ or CD8^+^ T cells were plated at 1*10^5/200 µl in co‐culture with macrophages plated at 1*10^4/well in 96‐well plates. CD3^+^ T cells were plated at 5*10^5/ml in co‐culture with macrophages plated at 1*10^5/well in 6‐well plates. MM cells were plated at 5*10^4/ml or 1*10^5/ml in 6‐well plates, according to effector‐to‐target ratio. T cells were activated with CD3/CD28 beads (Cat #11131D, Gibco, USA).

### 4.9 Flow Cytometry Antibodies

The following fluorochrome‐conjugated monoclonal antibodies were used for multicolor flow cytometry (BD Biosciences, USA): anti‐FAPα‐PE (#FAB3715B, RRID:AB_3126406, Novus, USA), anti‐PD‐L1‐APC (#329708, clone 29E.2A3, RRID:AB_940360), anti‐CD11b‐PerCP/Cyanine5.5 (#101228, clone ICRF44, RRID:AB_893232), anti‐CD14‐PE/Cy7 (#325618, clone HCD14, RRID:AB_830691), anti‐CD45‐PE/Cy7 (#304016, clone HI30, RRID:314404), anti‐CD38‐PerCP/Cyanine5.5 (#356614, clone HB‐7, RRID:AB_2562183), anti‐CD29‐Alexa Fluor488 (#303016, clone TS2116, RRID:AB_493025), anti‐CD138‐APC (#356506, clone MI15, RRID:AB_2561880), anti‐CD138‐FITC (#356508, clone MI15, RRID:AB_2561882), anti‐CD3‐FITC (#100204, clone UCHT1, RRID:AB_312661), anti‐CD4‐FITC (#344604, clone SK3, RRID:AB_1937227), anti‐CD4‐PerCP/Cyanine5.5 (#317418, clone OKT4, RRID:AB_571947), anti‐CD4‐PE (#300508, clone RPA‐T4, RRID:AB_314076), anti‐CD8‐FITC (#344704, clone SK1, RRID:AB_1877178), anti‐CD8‐PerCP/Cyanine5.5 (#301032, clone RPA‐T8, RRID:AB_893422), anti‐CD8‐APC (#344722, clone SK1, RRID:AB_2075388), anti‐PD‐1‐PE (#329906, clone EH12.2H7, RRID:AB_940483), anti‐CD28‐PerCP/Cyanine5.5 (#302921, clone CD28.2, RRID:AB_2073719), anti‐IFNγ‐PE/Cy7 (#502528, clone 4S.B3, RRID:AB_2123323), anti‐IL4‐APC (#500812, clone MP4.25D2, RRID:AB_315131), anti‐IL22‐PE/Cy7 (#366707, clone 2G12A41, RRID:AB_2571930), anti‐IL17‐APC (#512334, clone BL168, RRID:AB_2563986), anti‐IL9‐PE (#507605, clone MH9A4, RRID:AB_315487), anti‐IL21‐APC (#513008, clone 3A3‐N2, RRID:AB_11150407), anti‐IL10‐PE/Cy7 (#501420, clone JES3‐9D7, RRID:AB_2125385), anti‐Perforin‐PE/Cy7 (#308126, clone dG9, RRID:AB_2572049), anti‐GZMB‐Alexa Fluor 647 (#515406, clone GB11, RRID:AB_2566333), anti‐CD25‐APC (#356110, clone M‐A251, RRID:AB_2561977), anti‐Foxp3‐PE (#320208, clone 259D, RRID:AB_492982), from BioLegend USA. The data was analyzed with FlowJo X 10.0.7.

### 4.10 SA‐β‐Gal Assay

T cell senescence was assessed by detecting senescence‐associated β‐galactosidase (SA‐β‐Gal) activity using the Cellular Senescence Detection Kit‐SPiDER‐β‐Gal. Briefly, harvested T cells were stained with the SPiDER‐β‐Gal reagent according to the manufacturer's instructions. The stained cells were then analyzed using both fluorescence microscopy (Olympus IX71, Tokyo, Japan) and flow cytometry (BD Biosciences, USA). For microscopic observation, cell nuclei were counterstained with Cellstain‐Hoechst 33342 to facilitate cell identification and counting.

### 4.11 ELISA

The bone marrow supernatants of patients with different MM stages were collected, and different cytokines were detected as described by the respective manufacturers: Human M‐CSF AccuSignal ELISA Kit (Cat #KOA0253, Rockland, USA), Human Seprase/FAP AccuSignal ELISA Kit (Cat #KOA0627, Rockland, USA), Human TGFβ1 ELISA Kit (Cat #1117102) (All from Dakewe Bioengineering Co., Ltd, China).

### 4.12 Blocking Antibodies

To block human PD‐L1, PD‐1 or CTLA4 function in vitro, Ultra‐LEAF Purified anti‐human PD‐L1 antibody (4 µg/ml, Cat #329716, AB_11149168, BioLegend, USA), Ultra‐LEAF Purified anti‐human PD‐1 antibody (2 µg/mL, Cat #329926, AB_11147365, BioLegend, USA) or Ultra‐LEAF Purified anti‐human CTLA‐4 antibody (2 µg/mL, Cat #349931, AB_2783243, BioLegend, USA) was utilized. To block mouse PD‐L1 or PD‐1 function in vivo, Ultra‐LEAF Purified anti‐mouse PD‐L1 antibody (Cat #124339, AB_2800597, BioLegend, USA) or Ultra‐LEAF Purified anti‐mouse PD‐1 antibody (Cat #135248, AB_2783091, BioLegend, USA) was used. Anti‐mouse PD‐L1 or PD‐1 antibody was used at 150 µg per mouse every three days in mouse models.

### 4.13 Transcripts and Survival Analysis

The correlations of FAPα with progression‐free survival and overall survival were analyzed with GEPIA 3 (gepia3.bioinfoliu.com). The associations of FAPα expression with different microenvironment components across cancers were analyzed with Assistant for Clinical Bioinformatics (http://www.aclbi.com). The above analyses were based on The Cancer Genome Atlas (TCGA) data.

### 4.14 IHC Staining, IOD and Cells/HPF Analysis of Human Myeloma Bone Marrow Tissues and Mouse Models

Bone marrow biopsies from MM patients at different disease stages and tumor tissues from different mouse models were harvested for IHC. IOD was obtained with Quant Center, Pannoramic viewer (3D HISTECH, Hungary), and Image‐pro plus 6.0 (Media Cybernetics, Inc., Rockville, MD). Cells were counted in at least five randomly selected high‐power fields (HPF, 200x magnification), and the average number per HPF was calculated. The used antibodies were: anti‐human CD138 (Cat #BX500300, Biolynx, China), anti‐human CD68 (Cat #BX50031, AB_2936308, Biolynx, China), anti‐human FAP (Cat #BMS168, AB_10597443, eBioscience, USA), anti‐human/mouse CD138 (Cat #ab128936, AB_11150990, Abcam, USA), anti‐mouse F4/80 (Cat #70076, AB_2799771, Cell Signaling Technology, USA), anti‐mouse FAP (Cat #PA5‐99313, AB_2818246, Invitrogen, USA), anti‐mouse PD‐L1 (Cat #17952‐1‐AP, AB_10597552, Proteintech, USA), anti‐mouse CD4 (Cat #ab183685, AB_2686917, Abcam, USA), anti‐mouse CD8 (Cat #ab209775, AB_2860566, Abcam, USA), anti‐mouse IFNγ (Cat #105995‐T08, Sino Biological inc. China) and anti‐mouse GZMB (Cat #48298, Signalway Antibody, USA). The antibodies were diluted as recommended by the respective manufacturers.

### 4.15 Statistical Analysis

Data in bar graphs are presented as mean ± standard deviation (SD). Three independent experiments were performed. Statistical analyses were performed with GraphPad Prism 6.0 (GraphPad Software, San Diego, CA). The correlation between protein expression was analyzed by the Pearson correlation coefficient method. An unpaired or paired two‐tailed Student's *t*‐test was performed to compare two groups. The survival rate comparisons were made by the log‐rank test. A p value less than 0.05 was considered statistically significant. (*, *p* < 0.05; **, *p*＜0.01, ***, *p*＜0.001, ****, *p* < 0.0001 compared with the controls, respectively).

### 4.16 Study Approval

Written patient consent was provided, and ethics approval for human samples was granted by the Medical Ethics Committee of Zhejiang University School of Medicine (ethics approval IIT20210683A) for harvesting human tissues. All animal research was performed under a protocol approved by the Medical Experimental Animal Care Commission of Zhejiang University (ethics approval 2021895).

## Author Contributions

H.G. Z.D. and X. H. contributed to this work. H.G., J.H., Z.D., X.H., E.Z., W.L., and Z.C. designed the experiment. H.G., Z.D., X.D., and H.C. carried out animal studies. H.G., Z.D., and X.D. performed experiments and statistical analyses. E.Z., X.H., Z.D., X.D., H.C., W.C., J.Z., Y.H., H.Y., Y.Y., L.Y., Y.L., and J.H. helped to collect the clinical samples and analyze data. W.L. guided the writing. J.H., E.Z., Z.C., and W.L. guided and supervised the study.

## Conflicts of Interest

The authors declare no conflict of interest.

## Supporting information




**Supporting File 1**: advs73903‐sup‐0001‐SuppMat.docx.

**Supporting File 2**: advs73903‐sup‐0002‐DataFile.pdf.

## Data Availability

The data that support the findings of this study are available from the corresponding author upon reasonable request.
