# Supplementary material for: FAPα+ Macrophages Orchestrate Immune Evasion in Multiple Myeloma by Dual Regulation of PD‐L1 and T Cell Senescence
Source: Adv Sci (Weinh). 2026 Jan 30;13(18):e06239. doi: 10.1002/advs.202506239 (PMC13042965; doi:10.1002/advs.202506239)
Supplement: Supplementary file 1 — Supporting File 1: advs73903‐sup‐0001‐SuppMat.docx. [file ADVS-13-e06239-s001.docx]

**FAPα^+^ Macrophages Orchestrate Immune Evasion in Multiple Myeloma by Dual Regulation of PD-L1 and T Cell Senescence**

Huiyao Gu^1#^, Zhenfeng Dai^1#^, Xi Huang^1#^, Enfan Zhang^1^, Xinyuan Dai^1^, Haoguang Chen^1^, Wen Cao^1^, Jinna Zhang^1^, Yifan Hou^1^, Haimeng Yan^1^, Yang Yang^1^, Li Yang^1^, Yi Li^1^, Wenlong Lin^3*^, Zhen Cai^1,2*^ and Jingsong He^1,2*^

^1^Bone Marrow Transplantation Center, the First Affiliated Hospital, Zhejiang University School of Medicine, No.79 Qingchun Road, Hangzhou, 310002, Zhejiang, China

^2^Institute of Hematology, Zhejiang University, Hangzhou, 310058, Zhejiang, China

^3^Institute of Immunology and The Second Affiliated Hospital, Zhejiang University, Hangzhou, 310058, China

#These authors contributed equally to this work.

**Running Title:** FAPα promotes PD-L1 expression in myeloma-derived macrophages and induced T cell senescence.

**Significance:** A new subset of macrophages has been identified that can promote PD-L1 expression and accelerate T cell senescence to mediate tumor immune escape. This subset can be evaluated as a therapeutic target to improve immunotherapies in multiple myeloma.

**Keywords:** FAPα, Macrophage, PD-L1, Vimentin, Multiple myeloma, Immune Escape, Immunotherapy

^*^Address correspondence to: Jingsong He, The First Affiliated Hospital, School of Medicine, Zhejiang University, No.79, Qingchun Road, Hangzhou, 310002, China, Phone: 86.571.87236706, E-mail: [hejingsong@zju.edu.cn](mailto:hejingsong@zju.edu.cn)；Zhen Cai, The First Affiliated Hospital, School of Medicine, Zhejiang University, No.79, Qingchun Road, Hangzhou, 310002, China, Phone: 86.571.87236706, E-mail: [caiz@zju.edu.cn](mailto:caiz@zju.edu.cn)；Wenlong Lin, Zhejiang University, 866 Yuhang Tang Road, Hangzhou, Zhejiang, P. R. China. Phone: 86.0571.88981662; Email: [lwl210@foxmail.com](mailto:lwl210@foxmail.com).

**Supplementary Figures and legends**

Figure S1, related to Figure 2;

Figure S2, related to Figure 2;

Figure S3, related to Figure 3;

Figure S4, related to Figure 4;

Figure S5, related to Figure 5;

Figure S6, related to Figure 6;

Figure S7, related to Figure 7;

**
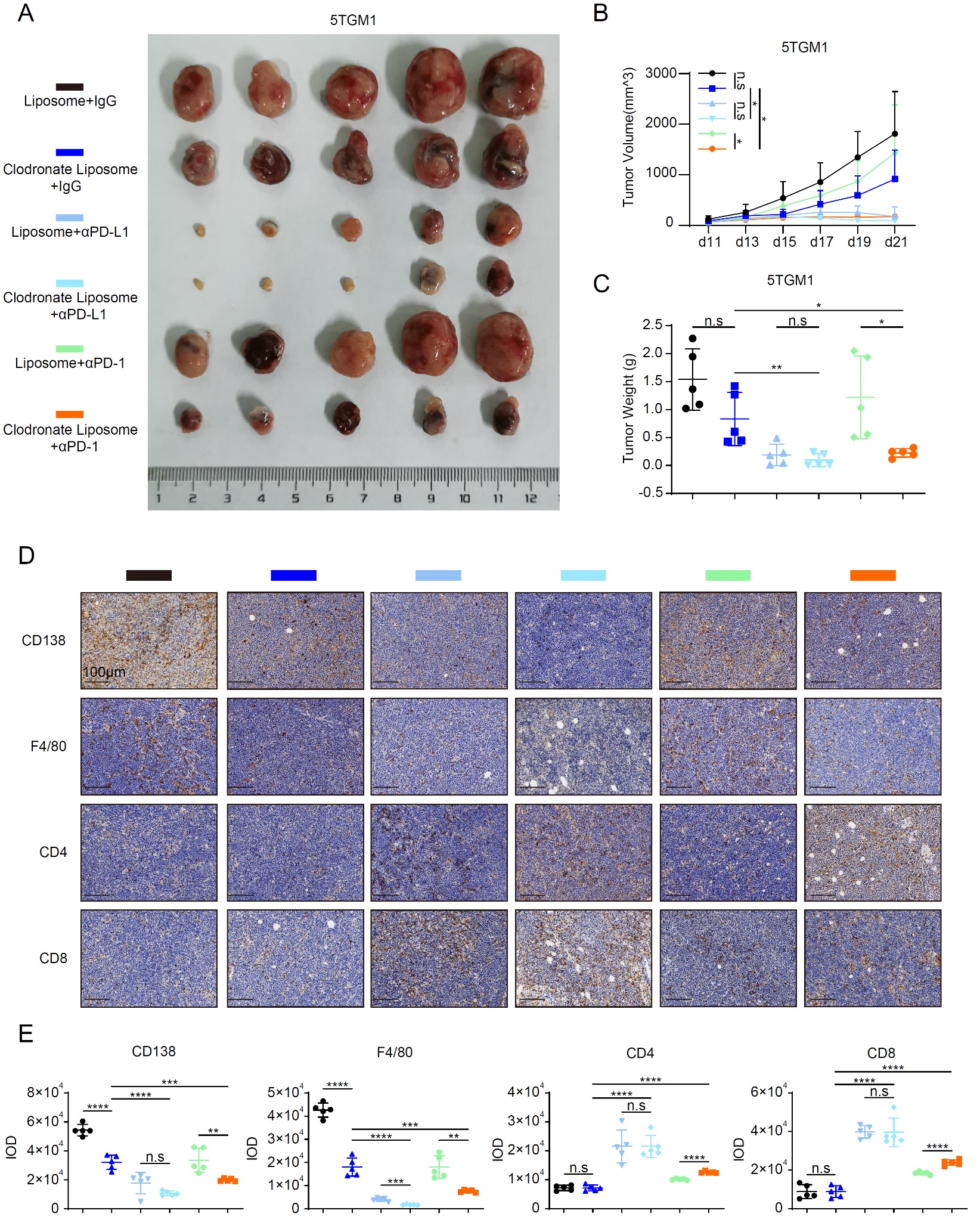
**

**Figure S1. Depletion of TAMs Potentiates the Efficacy of Anti-PD-1 Antibody Therapy.**

**(A)** Representative images of 5TGM1 tumors treated with clodronate liposomes ± αPD-L1/αPD-1 antibodies. **(B)** Tumor growth curves in the 5TGM1 model treated with clodronate liposomes ± αPD-L1/αPD-1 antibodies. Tumor volume was recorded every 2 days. **(C)** Tumor weight of 5TGM1 tumors treated with clodronate liposomes ± αPD-L1/αPD-1 antibodies. **(D)** IHC staining of 5TGM1 tumors from different treatment groups. **(E)** IOD analysis of CD138, F4/80, CD4, and CD8 in 5TGM1 tumors.

Data are presented as mean ± SEM. P values were determined by unpaired, two-sided t test. *P < 0.05, **P < 0.01, ***P < 0.001, ****P < 0.0001. Statistical analysis was performed using a 2-way ANOVA test in **B**, a 2-tailed Student’s t-test in **C and E**. TAMs, tumor-associated macrophages; PD-1, programmed cell death protein 1; PD-L1, programmed cell death ligand 1; IHC, Immunohistochemistry; IOD, Integrated Optical Density.

**
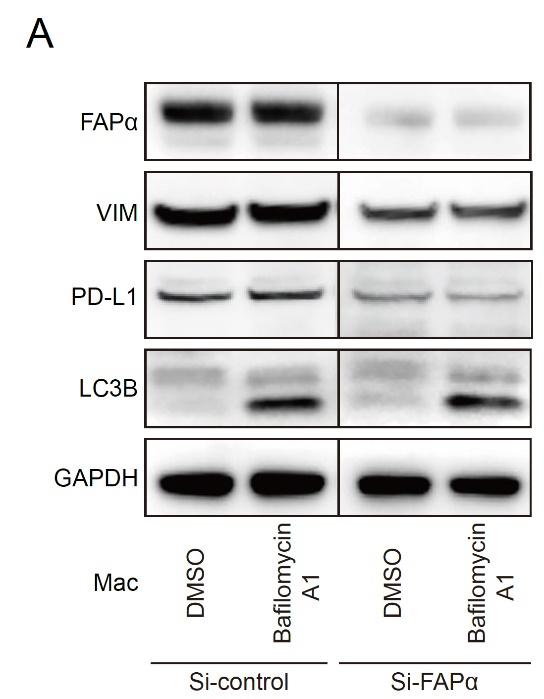
**

**Figure S2. FAPα do not Regulate PD-L1 via Autophagy.**

**(A)** WB analysis of macrophages treated with Bafilomycin A1 (100 nM, 12 h).


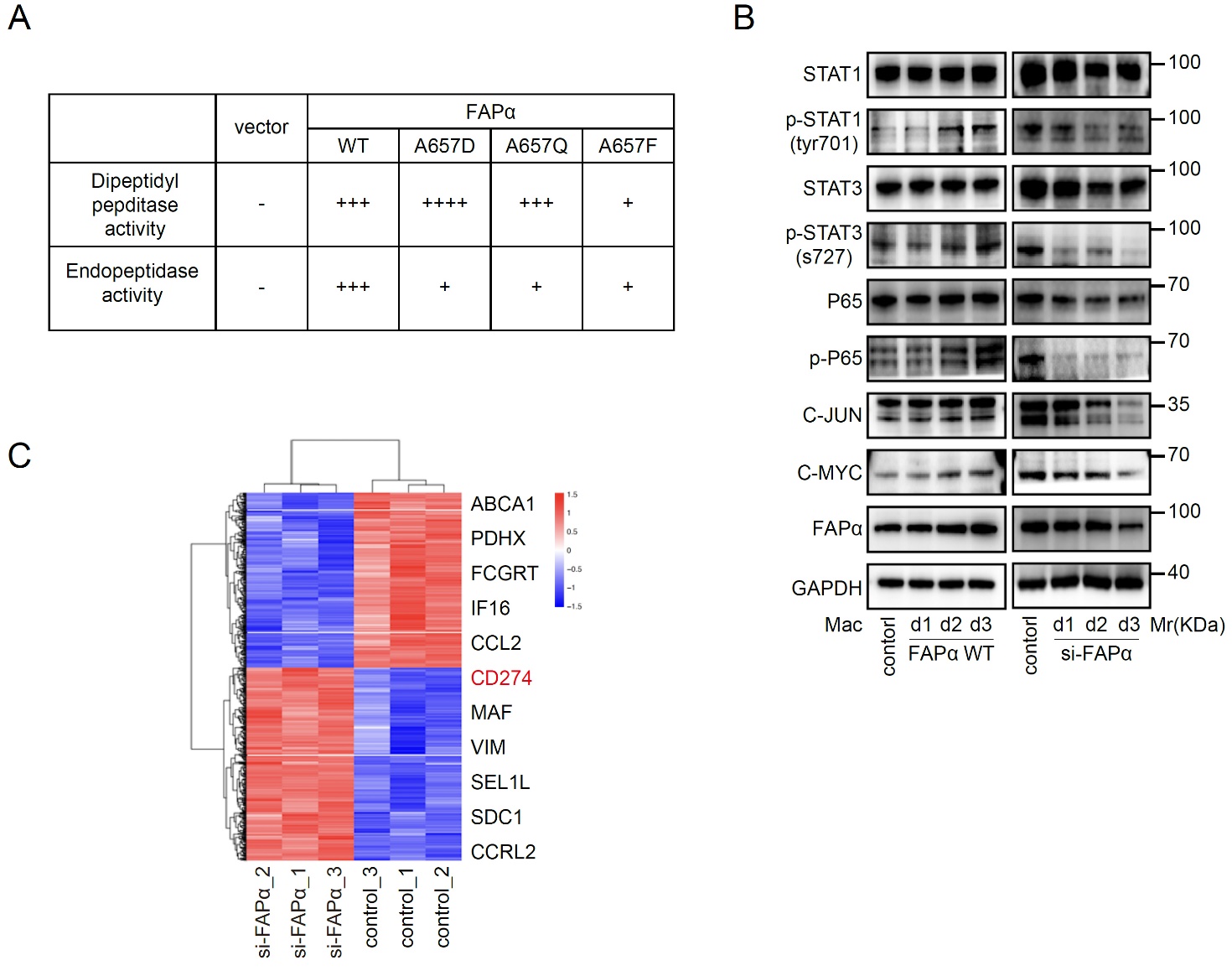


**Figure S3. Multiple Pathways Influenced by FAPα-Mediated PD-L1 Regulation.**

**(A)** Effects of different FAPα mutations (A657D, A657Q, and A657F) on enzymatic activity. **(B)** WB analysis of PD-L1-related signaling pathways in macrophages transfected with Si-FAPα or control siRNA. **(C)** Heatmap of differentially expressed genes in macrophages transfected with control siRNA or Si-FAPα.

Data are presented as mean ± SEM. FAPα, fibroblast activation protein alpha; PD-L1, programmed death-ligand 1; WB, Western blot; siRNA, small interfering RNA.

**
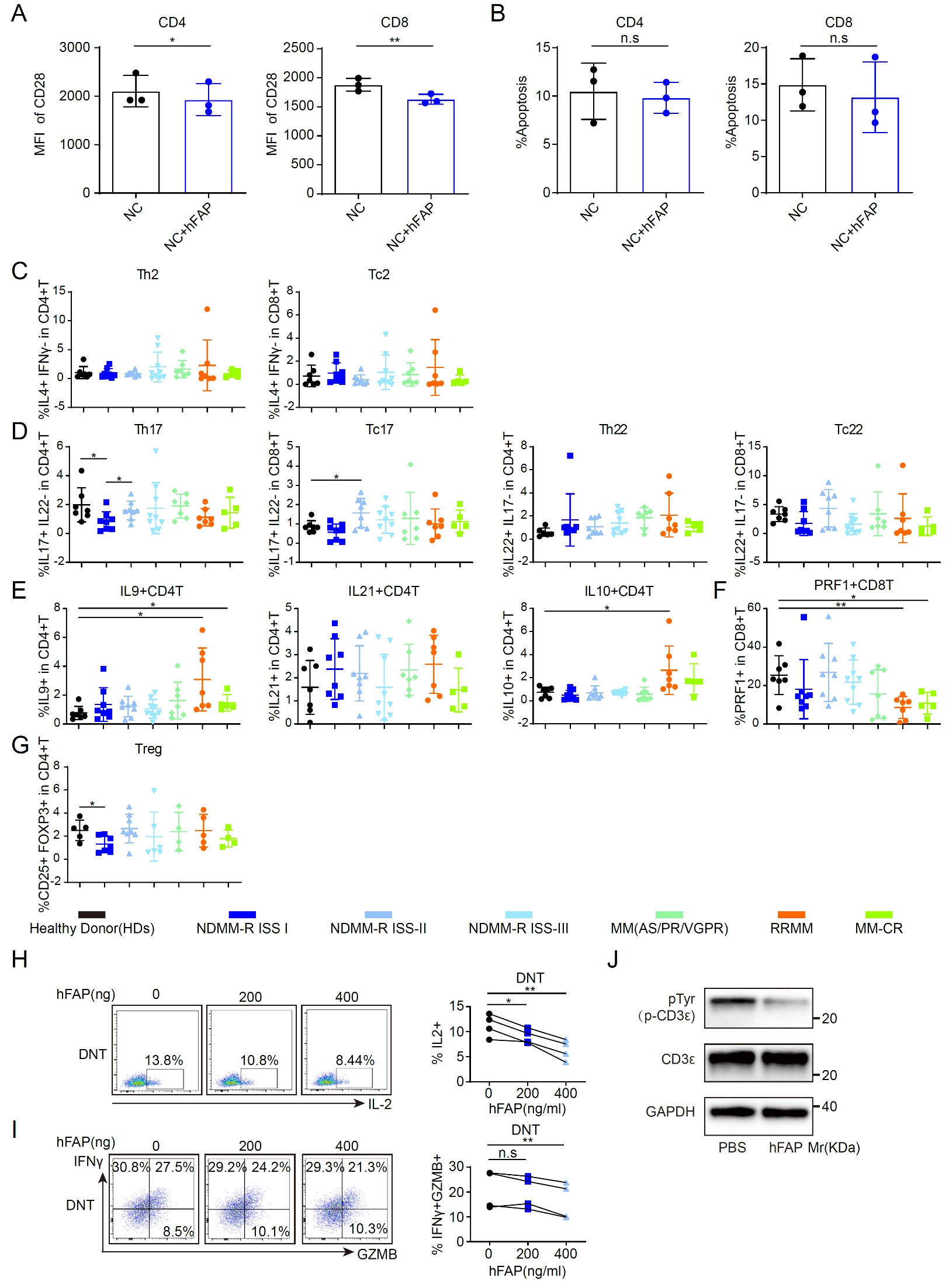
**

**Figure S4. Immune Microenvironment in MM Patients at Different Disease Stages.**

**(A)** Flow cytometry analysis of CD28 expression on T cells treated with FAPα. **(B)** Flow cytometry analysis of T cell apoptosis after treatment with FAPα. **(C)** IL-4 expression on T cells in BM samples from MM patients at different stages (n=51). **(D)** IL-17 and IL-22 expression on T cells in BM samples from MM patients at different stages (n=51). **(E)** IL-9, IL-21 and IL-10 expression on T cells in BM samples from MM patients at different stages (n=51). **(F)** Perforin expression on CD8^+^T cells in BM samples from MM patients at different stages (n=51). **(G)** Treg cell proportions in BM samples from MM patients at different stages (n=39). **(H-I)** Flow cytometry analysis of IL-2 (H), IFNγ^+^ GZMB^+^ (I) expression in DNT cells treated with FAPα. **(J)** WB analysis of the indicated key proteins of T cell signaling in Jurkat T cells treated with PBS or hFAPα.

Data are presented as mean ± SD. P values were determined by unpaired, two-sided t test. *P < 0.05, **P < 0.01, ***P < 0.001, ****P < 0.0001. Statistical analysis was performed using a 2-tailed Student’s t-test in **A-G, and I**.MM, multiple myeloma; BM, bone marrow; DNT, double-negative T cells; GZMB, granzyme B; IFNγ, Interferon-γ; Treg, regulatory T cells.


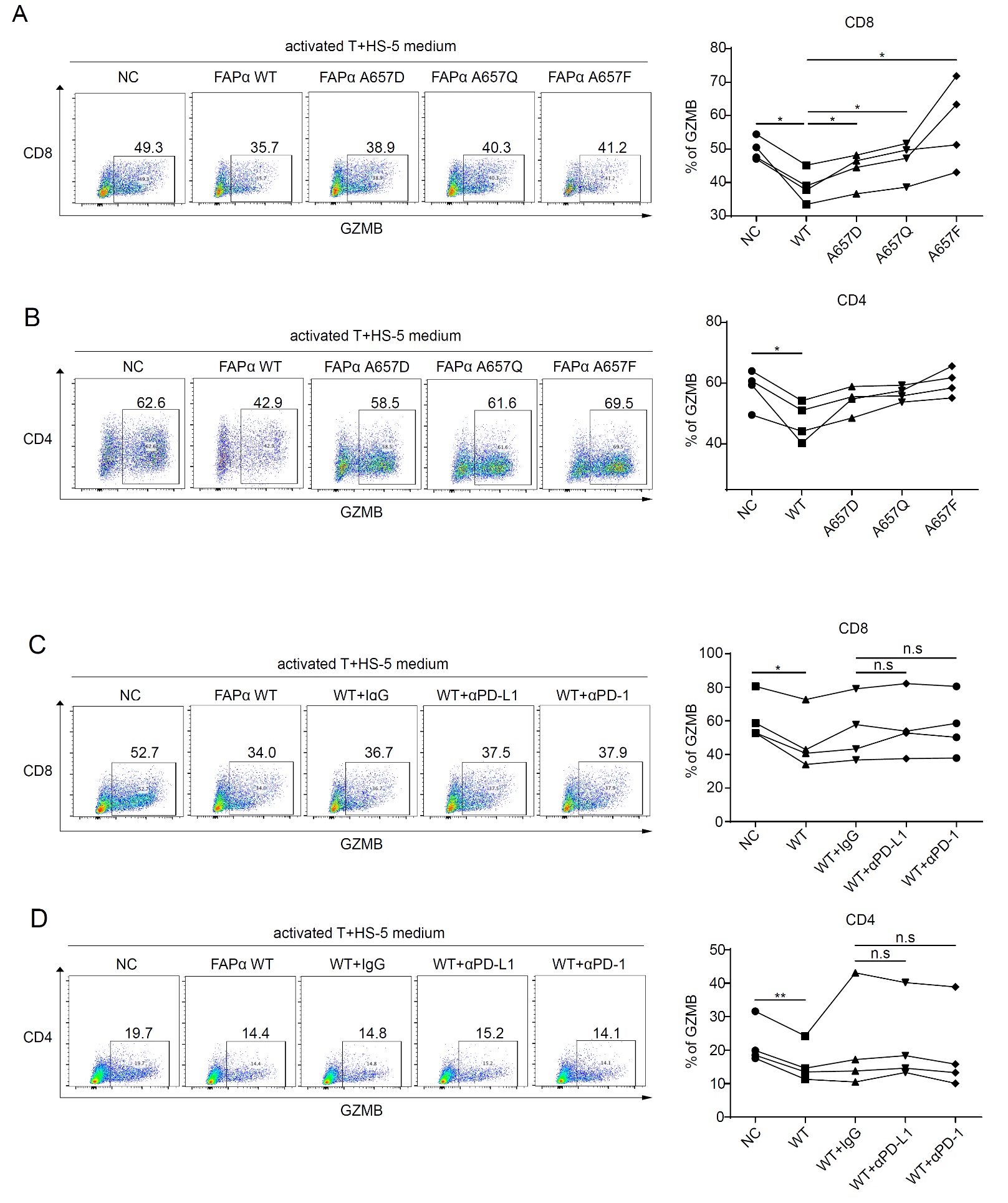


**Figure S5. The Effect of FAPα on T Cells is not Reversed by αPD-1/αPD-L1 Antibody Blockade.**

**(A-B)** GZMB expression in CD8^+^T **(A)** and CD4^+^T **(B)** cells treated with culture supernatant from HS-5 cells expressing WT or mutant FAPα (supernatant:medium=1:1). **(C-D)** GZMB expression in CD8 T **(C)** and CD4 T **(D)** cells incubated with or without αPD-1/αPD-L1 antibodies under culture supernatant from HS-5 cells expressing FAPα WT.

Data are presented as mean ± SD. Statistical analysis was performed using a 2-tailed Student’s t-test in **A-D**. *P < 0.05, **P < 0.01. FAPα, fibroblast activation protein alpha; PD-1, programmed cell death protein 1; PD-L1, programmed death-ligand 1; IFNγ, Interferon-γ; GZMB, granzyme B.


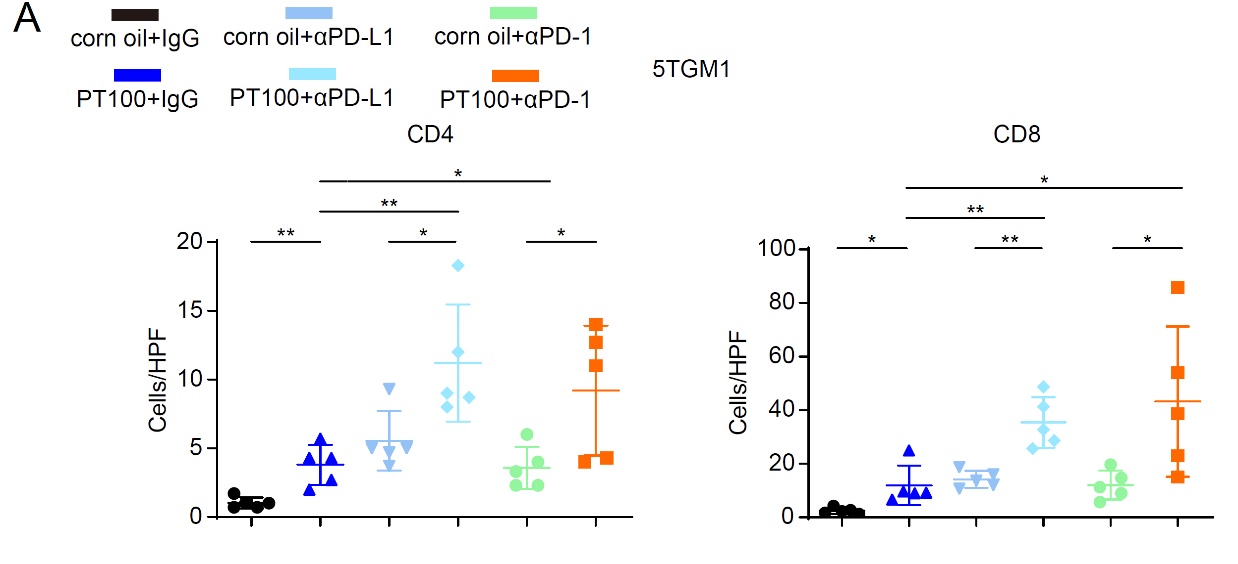


**Figure S6. Combination Therapy Enhances T Cell Infiltration in 5TGM1 Tumor Models.**

**(A)** Quantification of CD4^+^ T and CD8^+^ T cell infiltration (cells per high-power field) by IHC analysis in 5TGM1 tumor model.

Data are presented as mean ± SD. Statistical analysis was performed using a 2-tailed Student’s t-test in **A**. *P < 0.05, **P < 0.01.IHC, Immunohistochemistry.


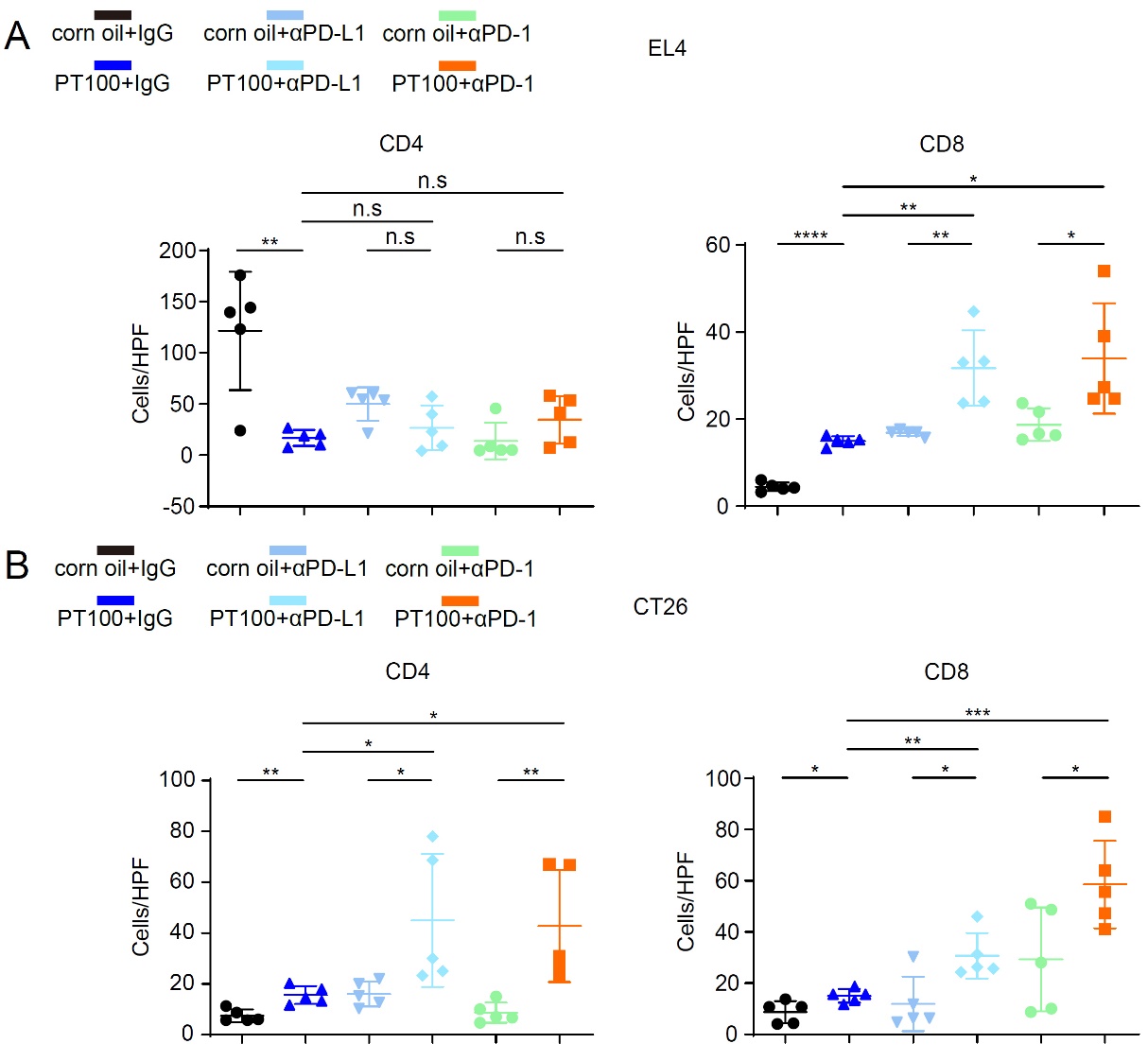


**Figure S7. Combination Therapy Enhances T Cell Infiltration in other Tumor Models.**

**(A-B)** Quantification of CD4^+^ T and CD8^+^ T cell infiltration (cells per high-power field) by IHC analysis in EL4 **(A)** and CT26 **(B)** tumor model.

Data are presented as mean ± SD. Statistical analysis was performed using a 2-tailed Student’s t-test in **A and B**. *P < 0.05, **P < 0.01, ***P < 0.001. IHC, Immunohistochemistry.


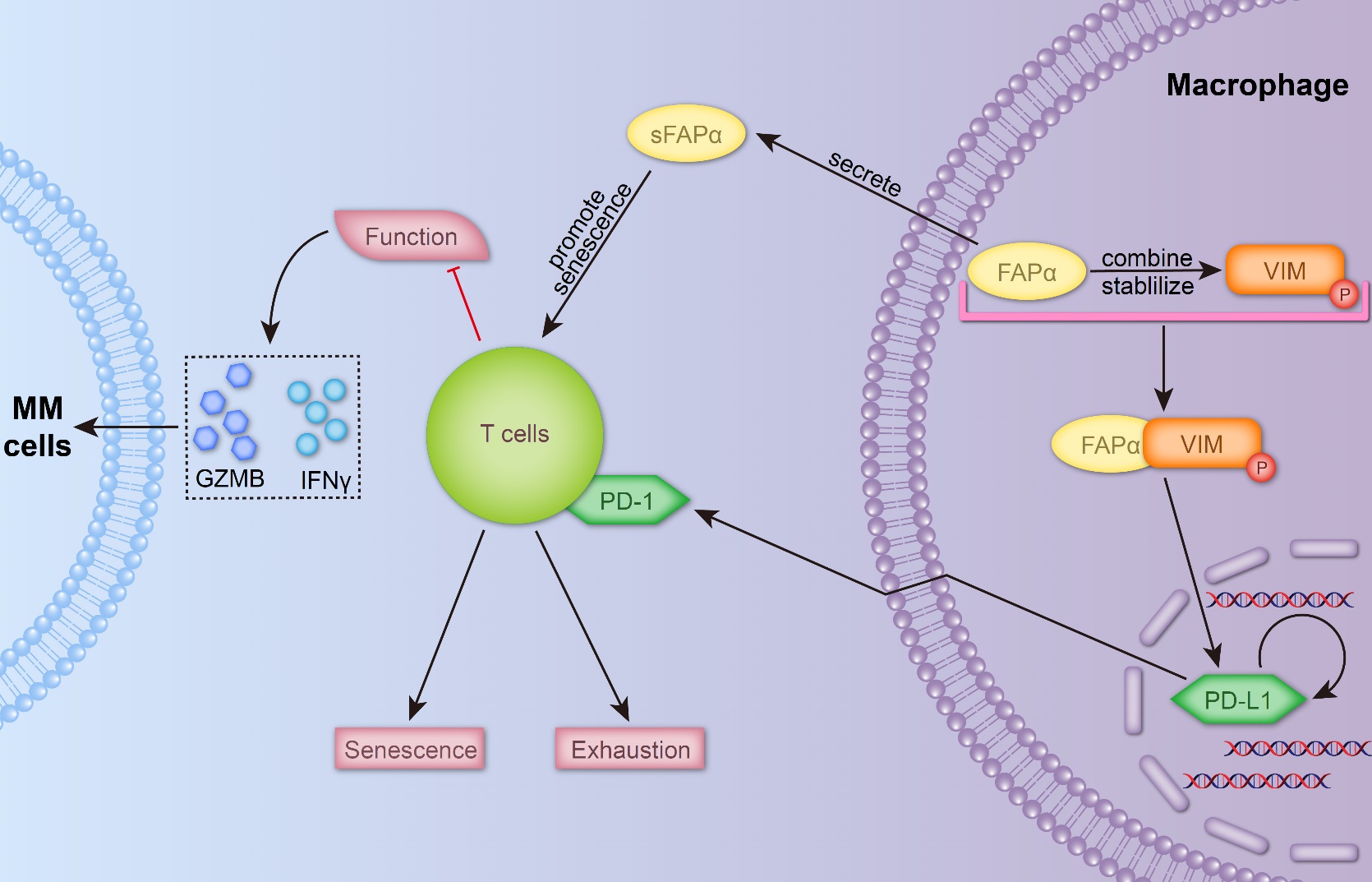


**Figure S8. Schematic Diagram of FAPα^+^ Macrophages Orchestrate** **Immune Evasion in Multiple Myeloma by Dual Regulation of PD-L1 and T Cell Senescence.** On the one hand, FAPα interacts with vimentin (VIM) and promotes its phosphorylation at the S72 residue, thereby inducing the expression of PD-L1. On the other hand, FAPα maintains PD-L1 protein stability by preserving its N-glycosylation modification and inhibiting proteasomal degradation. FAPα⁺ macrophages accelerate T cell senescence via the secretion of soluble FAPα protein, resulting in immune evasion of multiple myeloma.
