# Supplementary material for: FAPα+ Macrophages Orchestrate Immune Evasion in Multiple Myeloma by Dual Regulation of PD‐L1 and T Cell Senescence
Source: Adv Sci (Weinh). 2026 Jan 30;13(18):e06239. doi: 10.1002/advs.202506239 (PMC13042965; doi:10.1002/advs.202506239)

Figure 1 J

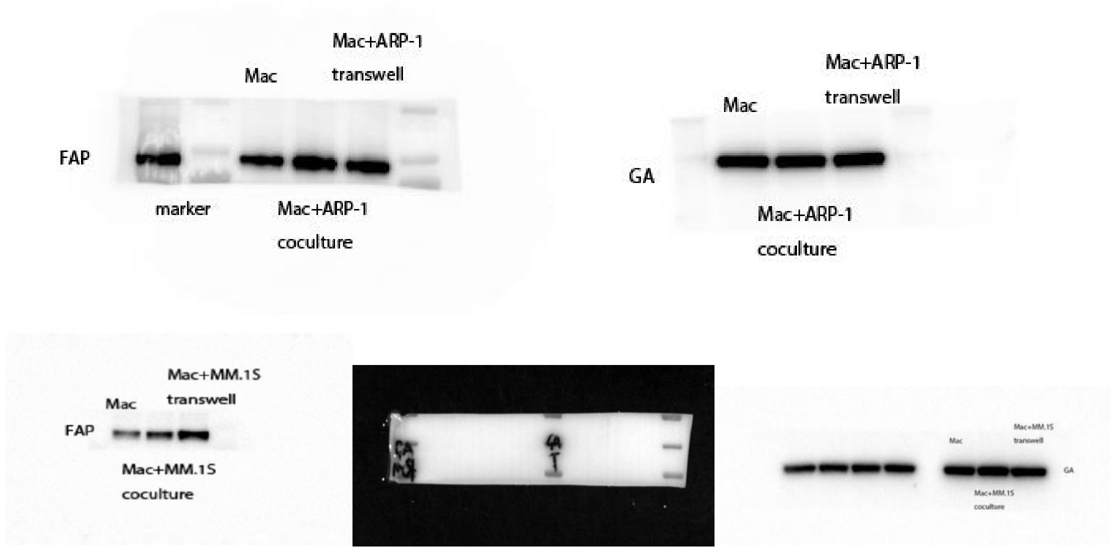


Figure 1 L

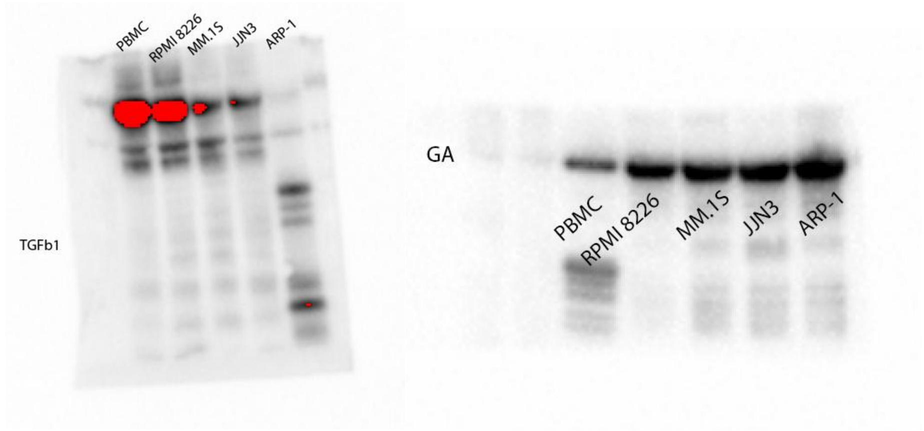


Figure 1 M

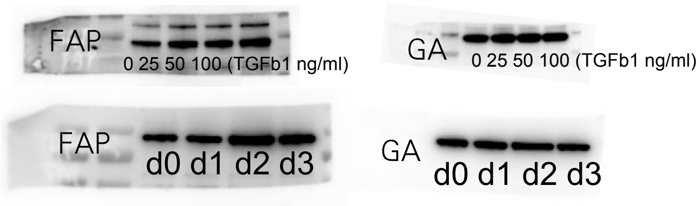

Figure 2 B

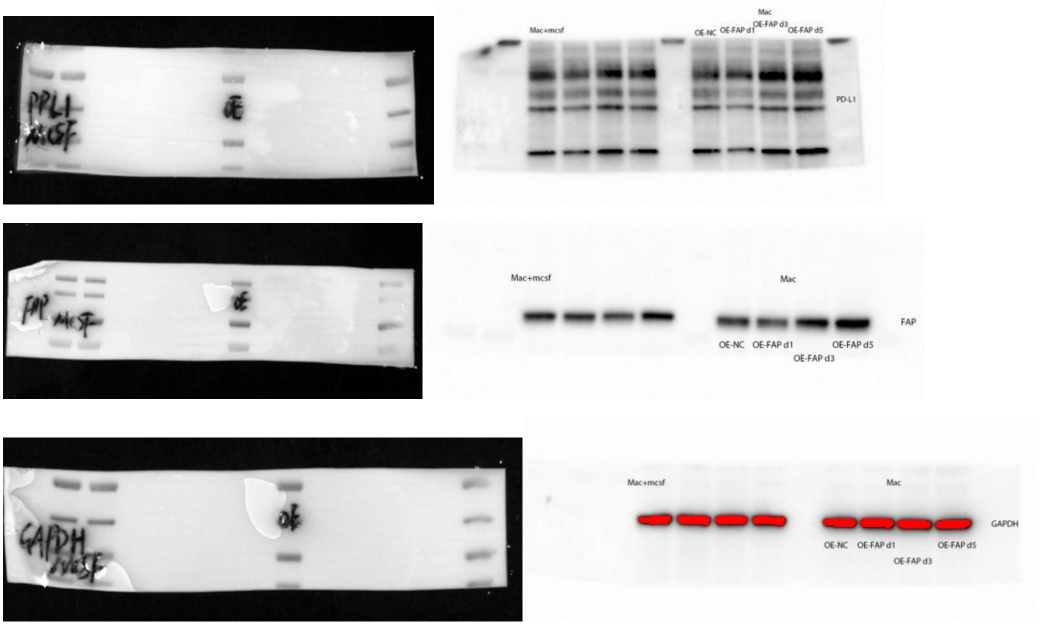


Figure 2 C

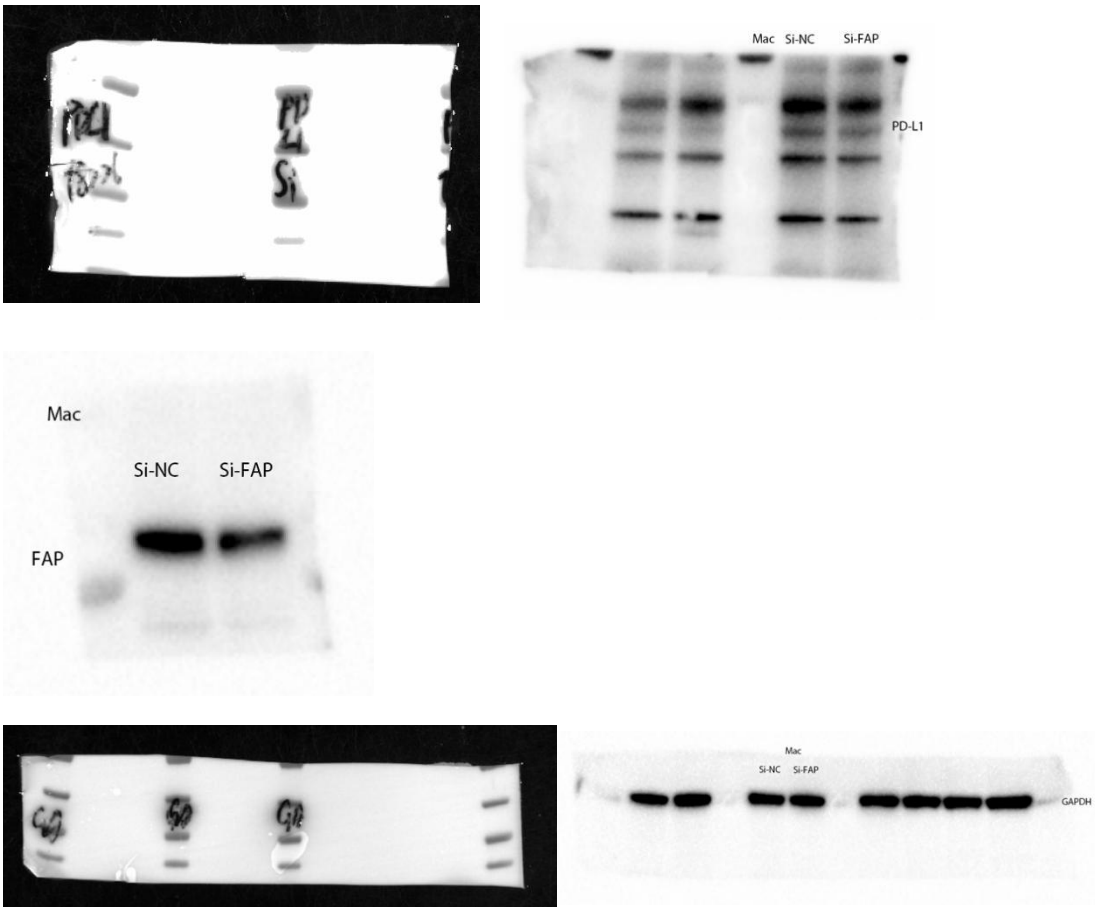

Figure 2 E

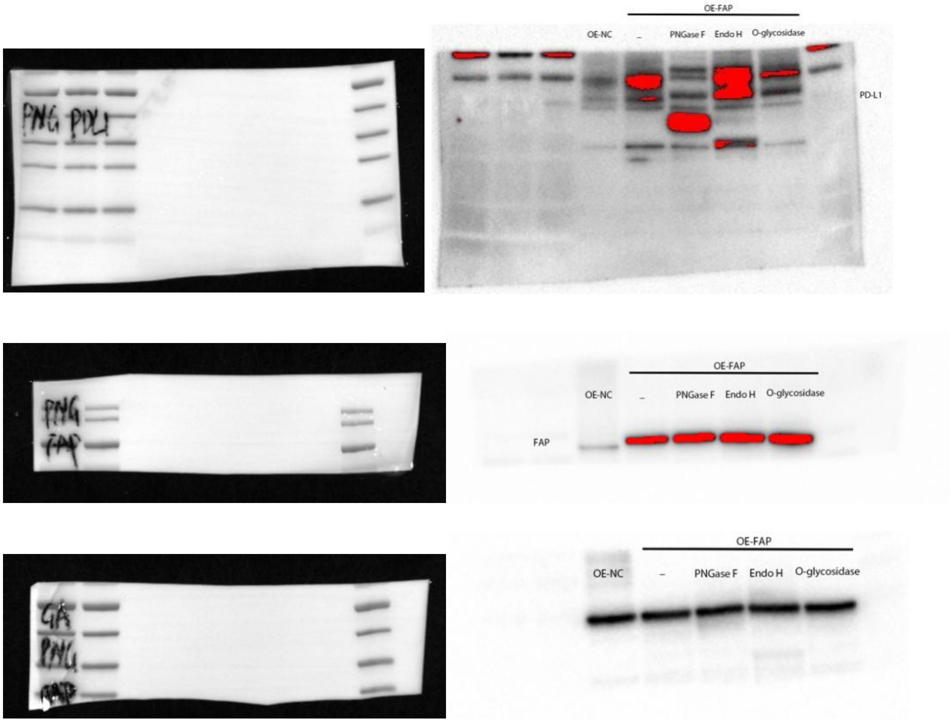


Figure 2 F

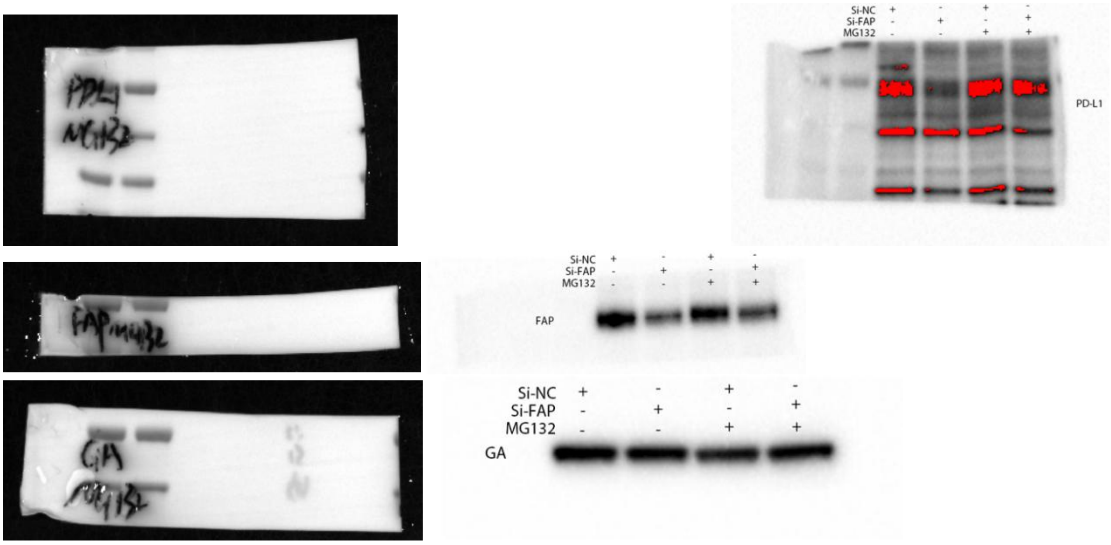

Figure 2 G

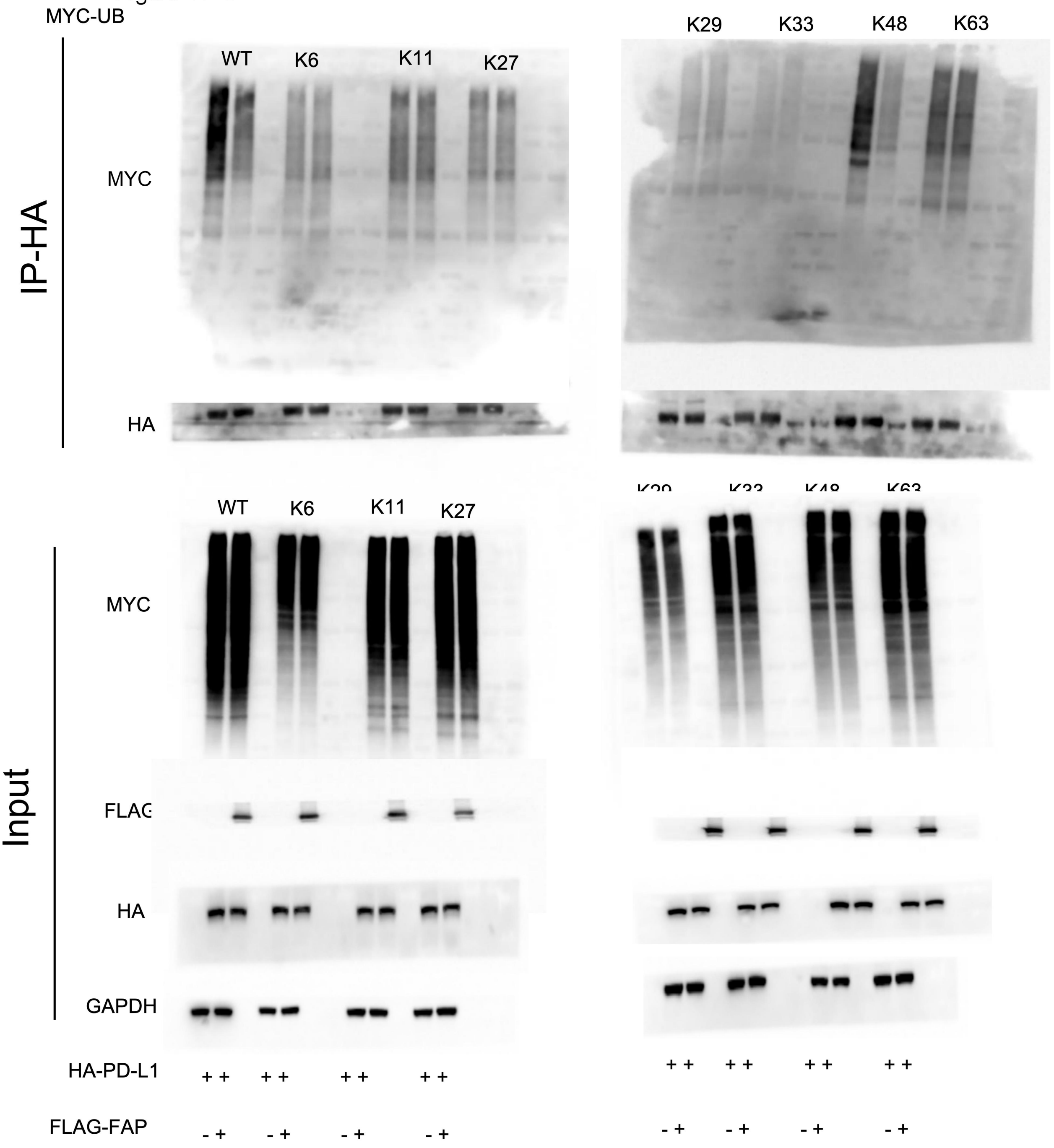
MYC-UB
WT
K6
K11
K27
K29
K33
K48
K63
IP-HA
MYC
HA
Input
MYC
FLAG
HA
GAPDH
HA-PD-L1
+ + + + + + + +
+ + + + + + + +
FLAG-FAP
- + - + - + - +
- + - + - + - +

Figure 3D, Figure 3F

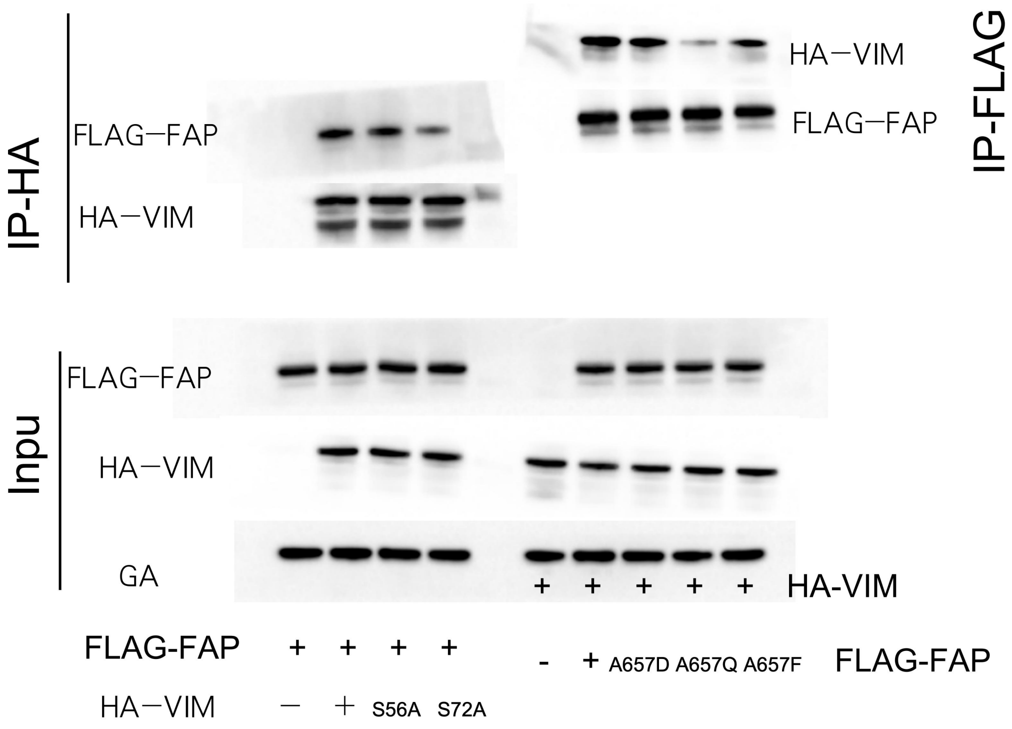


Figure 3E

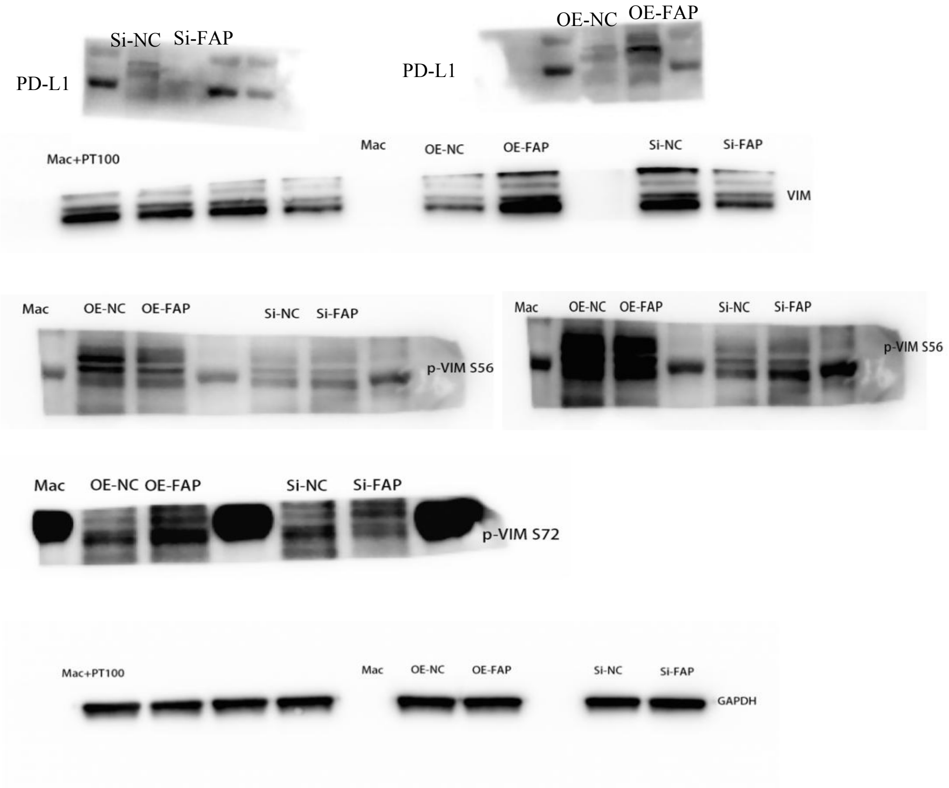

Figure 3G

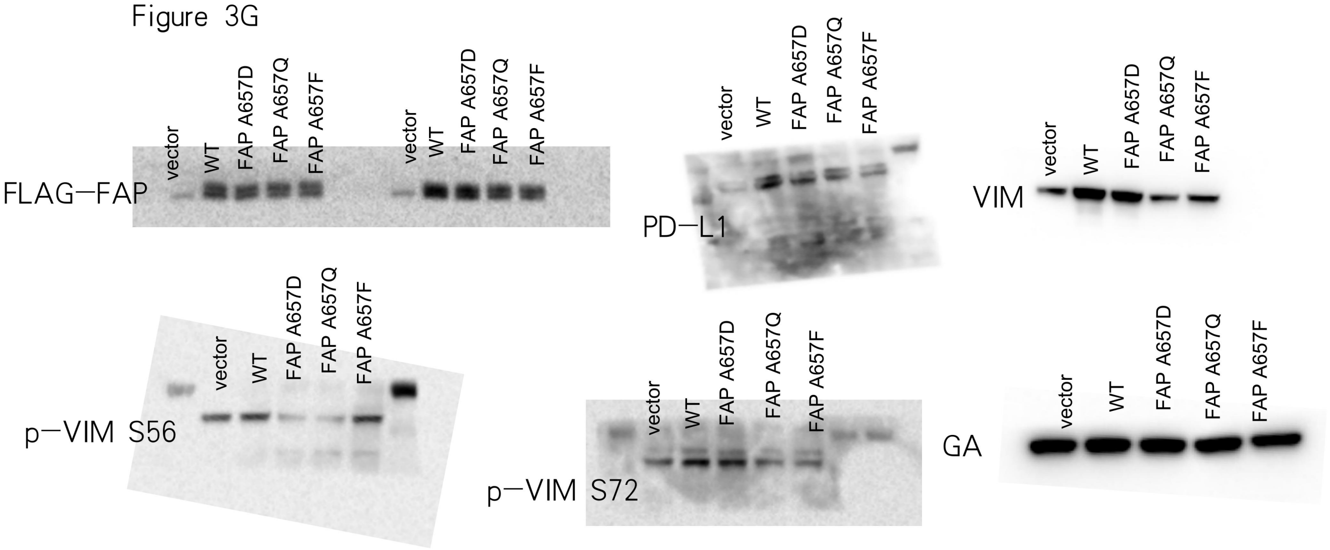


Figure 3H

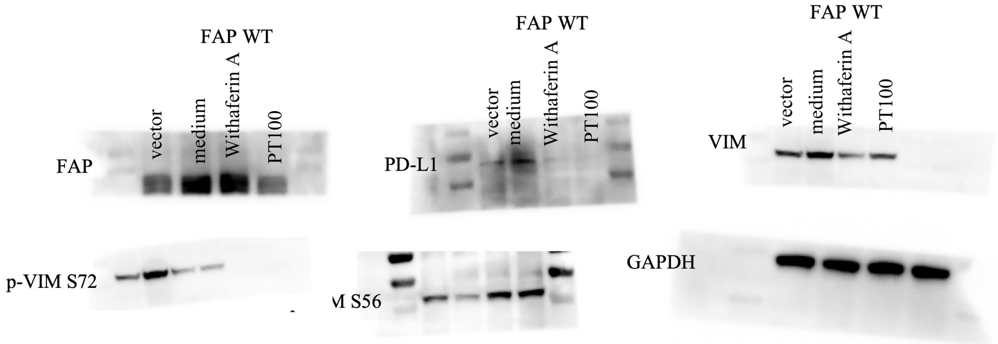


Figure5 C

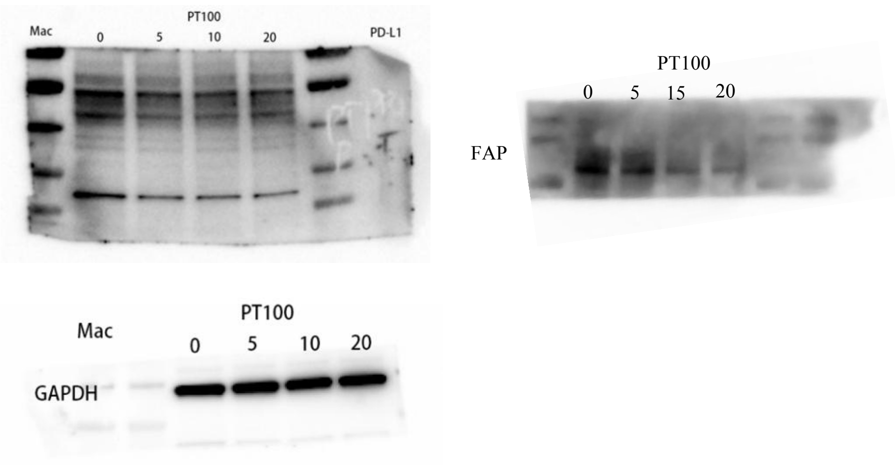

## Supplementary figure 2 A

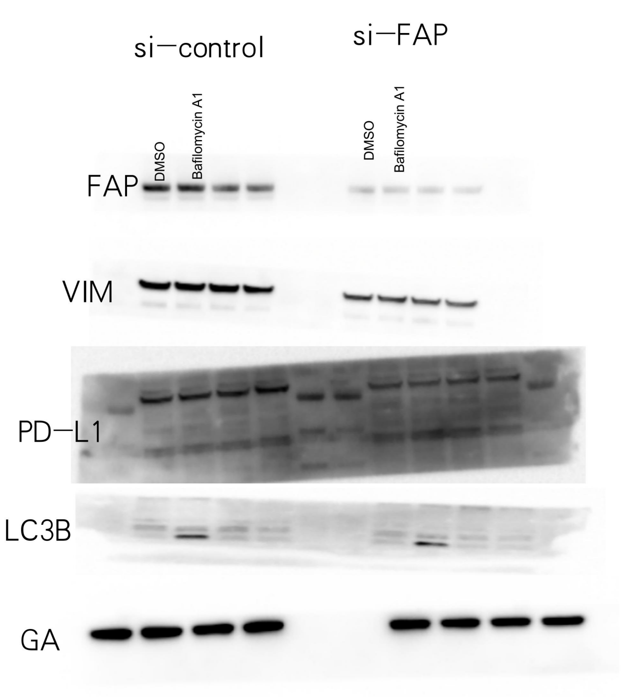


## Supplementary figure 2 C

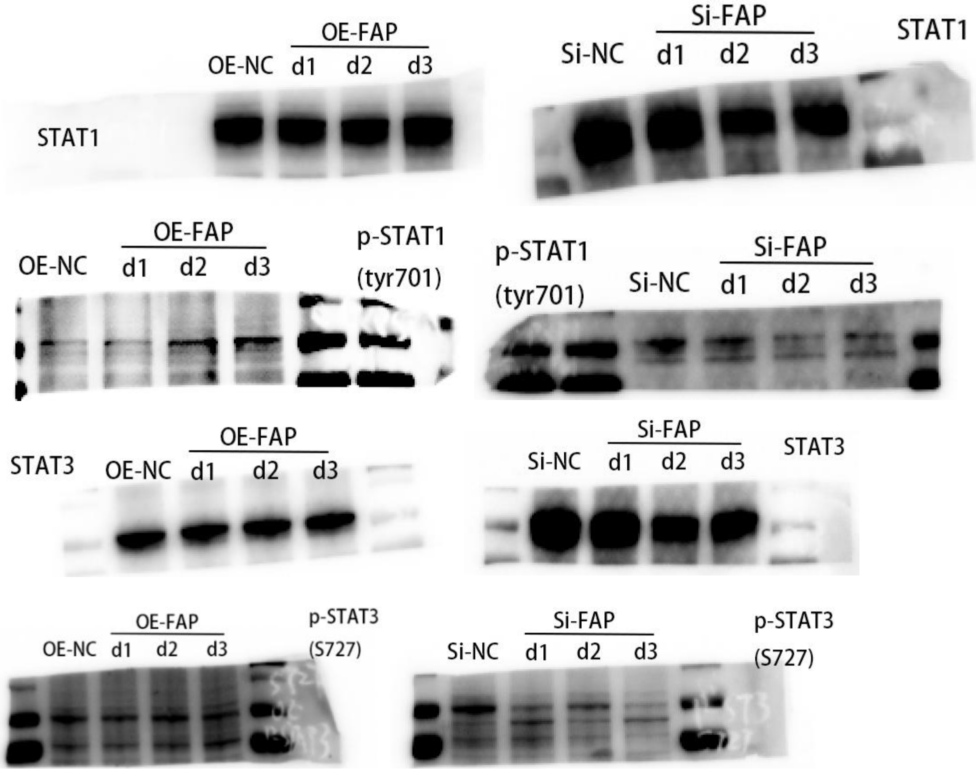

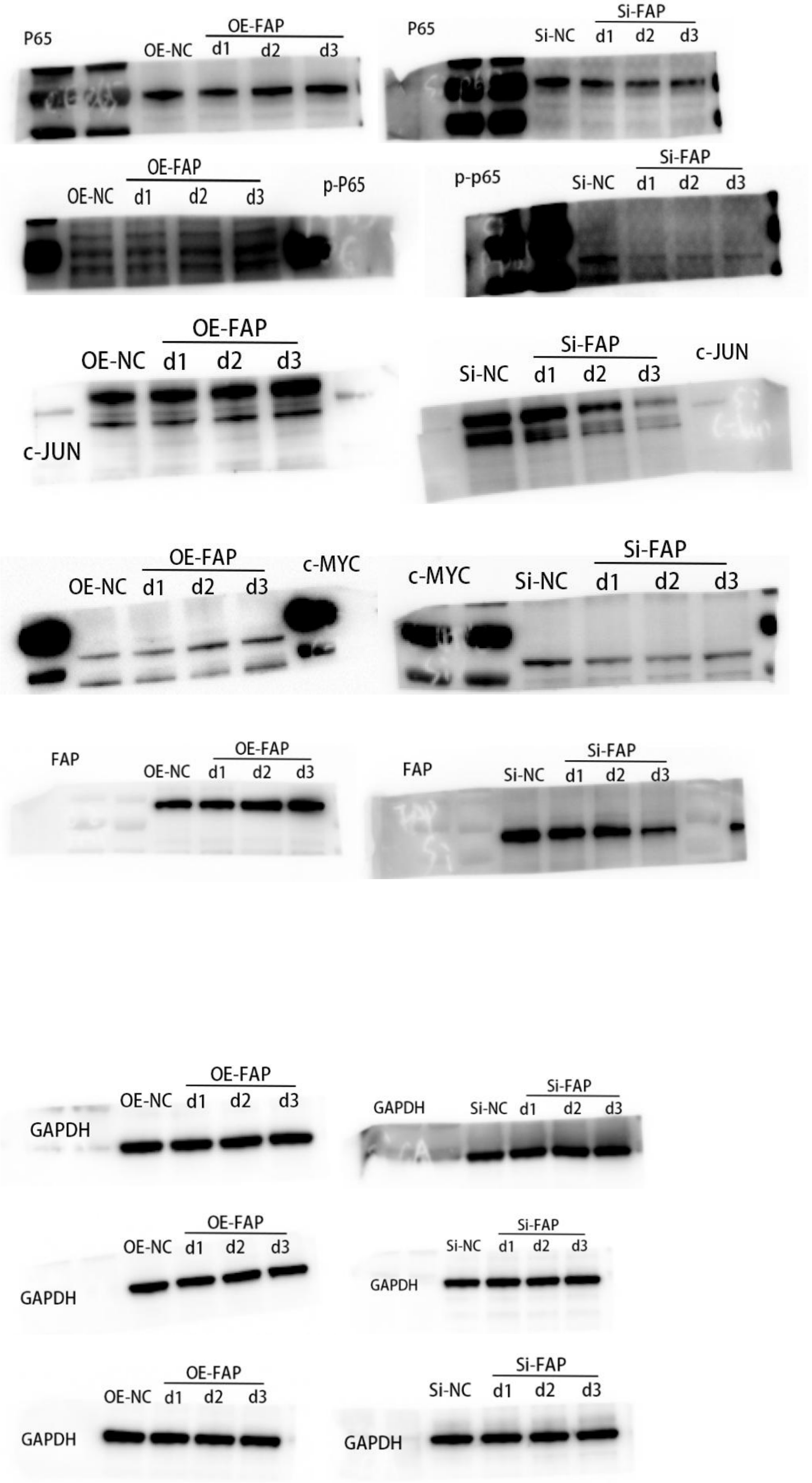


Supplementary figure 4 J

Jurkat

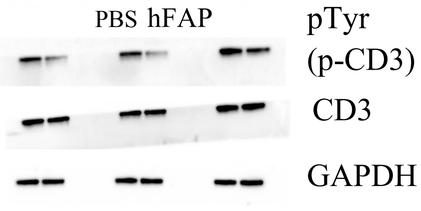

Supplement: Supplementary file 2 — Supporting File 2: advs73903‐sup‐0002‐DataFile.pdf. [file ADVS-13-e06239-s002.pdf]
